# Extensive Co-Operation between the Epstein-Barr Virus EBNA3 Proteins in the Manipulation of Host Gene Expression and Epigenetic Chromatin Modification

**DOI:** 10.1371/journal.pone.0013979

**Published:** 2010-11-15

**Authors:** Robert E. White, Ian J. Groves, Ernest Turro, Jade Yee, Elisabeth Kremmer, Martin J. Allday

**Affiliations:** 1 Section of Virology, Imperial College London, London, United Kingdom; 2 Department of Epidemiology and Biostatistics, Imperial College London, London, United Kingdom; 3 Institute of Molecular Immunology Helmholtz Zentrum München - German Research Center for Environmental Health, Munich, Germany; Karolinska Institutet, Sweden

## Abstract

Epstein-Barr virus (EBV) is able to drive the transformation of B-cells, resulting in the generation of lymphoblastoid cell lines (LCLs) *in vitro*. EBV nuclear proteins EBNA3A and EBNA3C are necessary for efficient transformation, while EBNA3B is dispensable. We describe a transcriptome analysis of BL31 cells infected with a series of EBNA3-knockout EBVs, including one deleted for all three EBNA3 genes. Using Affymetrix Exon 1.0 ST microarrays analysed with the MMBGX algorithm, we have identified over 1000 genes whose regulation by EBV requires one of the EBNA3s. Remarkably, a third of the genes identified require more than one EBNA3 for their regulation, predominantly EBNA3C co-operating with either EBNA3B, EBNA3A or both. The microarray was validated by real-time PCR, while ChIP analysis of a selection of co-operatively repressed promoters indicates a role for polycomb group complexes. Targets include genes involved in apoptosis, cell migration and B-cell differentiation, and show a highly significant but subtle alteration in genes involved in mitosis. In order to assess the relevance of the BL31 system to LCLs, we analysed the transcriptome of a set of EBNA3B knockout (3BKO) LCLs. Around a third of the genes whose expression level in LCLs was altered in the absence of EBNA3B were also altered in 3BKO-BL31 cell lines.

Among these are *TERT* and *TCL1A*, implying that EBV-induced changes in the expression of these genes are not required for B-cell transformation. We also identify 26 genes that require both EBNA3A and EBNA3B for their regulation in LCLs. Together, this shows the complexity of the interaction between EBV and its host, whereby multiple EBNA3 proteins co-operate to modulate the behaviour of the host cell.

## Introduction

Epstein-Barr virus (EBV) is ubiquitous in the human population, with up to 95% of humans asymptomatically infected. *In vitro*, EBV can very efficiently induce the activation and continuous proliferation of resting human B cells. The resulting lymphoblastoid cell lines (LCLs) carry the viral genome as extra-chromosomal episomes and express only nine ‘latent’ EBV proteins in the latency III or ‘growth program’ of viral gene expression. This program is comprised of six nuclear antigens (EBNAs 1, 2, 3A, 3B, 3C & LP), three membrane associated proteins (LMP1, LMP2A & 2B) and also several untranslated RNA species [1], [2]. The uncontrolled proliferation driven by latency III is also seen transiently *in vivo*. In the absence of effective cytotoxic T-cell surveillance, an aggressive B-lymphoproliferative disease (post-transplant lymphoproliferative disease; PTLD) or, rarely, malignant lymphomas may arise [3].

In humans, it is now considered probable that to establish persistence, EBV initially infects resting (naïve) B cells and drives these to proliferate as activated B-blasts, much as occurs *in vitro*. Infected B-blasts then migrate into germinal centres and differentiate to eventually become resting memory B cells [4], [5]. This differentiation process is coupled to a progressive shut-down of EBV gene expression from the growth-promoting latency III state achieved *in vitro*, to the latency II program seen in germinal centre cells [6] and ending in the establishment of resting memory B cells in which no latent proteins are expressed.

Generally only expressed during latency III, EBNA3A, EBNA3B and EBNA3C comprise a protein family with no known homologues outside the primate lymphocryptoviruses. They share a gene structure (each having a short 5′ exon and longer 3′ exon) and occur as a tandem array in the EBV genome, having a limited amino acid sequence homology. Together with the other EBNAs, the EBNA3 transcripts are alternatively spliced from very long mRNAs generally initiated at the Cp latency promoter; LCLs have only a few copies of these transcripts per cell, suggesting their expression is tightly regulated and the turnover of the EBNA3s is slow [7].

Along with EBNA2 and LMP1, EBNA3A and EBNA3C were identified as essential for the transformation of B cells into LCLs [8], [9], although more recently an EBNA3A-deleted EBV has been used to transform B cells, albeit with reduced efficiency. It seems likely that EBNA3A (acting with EBNA3C) facilitates LCL outgrowth through the epigenetic repression of the p16^INK4A^ gene that would otherwise cause arrest or senescence via the retinoblastoma (Rb) pathway [10], [11]. In contrast, EBNA3B is entirely dispensable for generating LCLs [12], [13].

EBNA2, the major transcriptional activator of latency III, is primarily directed to genes by its binding to the DNA-binding factor CBF1 (RBPJk) [14]. All the EBNA3 proteins also bind to the same region of CBF1, can all inhibit EBNA2-mediated activation of the LMP2 and Cp promoters [15], [16], [17], [18], [19]. Additionally, all of the EBNA3s exhibit robust repressor activity when targeted directly to DNA [18], [20], [21], [22] . They are all known to interact with one or more cellular factors involved in transcriptional repression or silencing, including histone deacetylases (HDACs) and C-terminal binding protein (CtBP) [21], [23], [24], [25].

We have recently explored the mechanism of transcriptional repression of both the pro-apoptotic protein Bim and the cell cycle inhibitor p16^INK4A^ by EBNA3A and EBNA3C. In both cases the EBNA3-dependent epigenetic change associated with repression is the trimethylation of lysine 27 on histone H3 (H3K27Me3) [11], [26]. In the case of p16^INK4A^, this is dependent on the binding of EBNA3A and EBNA3C to CtBP [11]. This epigenetic modification is established and repression is maintained by the action of the polycomb group of proteins (PcG) [27].

In addition to their role in transcriptional repression, EBNA3A and EBNA3C are known to play a role in the regulation of the cell cycle. A number of studies assert that EBV overrides or modifies cell cycle checkpoints in G1, G2 and mitosis (reviewed in O'Nions and Allday [28]). Consistent with the ability to override cell cycle checkpoints, it has been observed that genomic instability is increased by latent EBV [29], and is probably attributable in part to the ability of EBNA3C to override a mitotic spindle checkpoint [30].

EBNA3C and EBNA3A are able to co-operate with oncogenic ras (Ha-ras) in transforming embryonic rat fibroblasts (which again requires CtBP interaction), and when over expressed EBNA3C can overcome a mitotic checkpoint [21], [31], [32]. Additionally, EBNA3C has been reported to bind to a number of cell-cycle regulators, including cyclin A, SCF^SKP2^, pRb, Myc, p53 and MDM2 [24], [33], [34], [35], [36], [37], [38].

In order to understand the transcriptional targets of the EBV proteins, a number of microarray studies have now been undertaken to investigate their transcriptional targets. However, these studies are limited by their narrow scope, typically focusing on individual proteins outside the context of other EBV gene products, and being undertaken in a wide range of different cell backgrounds. As a consequence, the agreement between studies is typically very limited (reviewed by Calderwood and Johannsen [39]). In contrast to EBNA2 and LMP1, there are few published microarray studies looking at the impact of the EBNA3s on host gene expression [10], [40], [41]. Therefore in order to properly understand the interaction of the EBNA3 proteins with host cells, we have generated independent knockouts of EBNA3A, EBNA3B and EBNA3C in the context of the B95-8 BAC system [42]. A similar approach has been used to investigate mutants of EBNA3A [10], and to generate a mutant lacking EBNA3B which is impaired for EBNA3C expression [40].

Since EBNA3C is essential for immortalisation of B-cells, we cannot produce LCLs with all of our virus mutants. However, *de novo* infection of EBV-negative BL cells results in the latency III gene expression profile which produces a phenotypic alteration of the cell to a more LCL-like phenotype [43], [44]. We have therefore adopted the BL31 cell line as a platform for comparing the function of various EBV proteins, by generating cell lines infected by various EBV mutants. To maximise the benefit of this system, we have undertaken a transcriptional analysis using the Affymetrix Exon 1.0 ST microarray platform on the various virus mutants, which has probes for almost all human genes. This study demonstrates the value of a co-ordinated approach to assessing the roles of the EBV genes and proteins, showing extensive co-operation between the EBNA3s in altering host gene transcription, and identifying a number of genes consistently modulated by EBV, which may play key roles in effecting the biological imperatives of the virus.

## Results

### Microarray strategy

We have previously reported the generation of mutant EBVs lacking either the EBNA3A, 3B or 3C gene, and the production of BL31 cell lines carrying these mutants [42]. These mutants were generated in the EBV-BAC (or Maxi-EBV) system [45] and revertants (where the targeted mutation has been restored to wild-type sequence) generated for each EBV mutant. We have additionally constructed a virus lacking the entire EBNA3 locus (E3KO), and its revertant (E3rev). BL31 cells infected with E3KO grew out poorly and unlike the single gene knockouts reported previously, the cells retain a requirement for supplements (α-thioglycerol and sodium pyruvate) in the medium. In order to make valid comparisons between the wild-type-infected, knockouts, and parental BL31 cells, we therefore generated a further set of cell lines which we grew out in the presence of the supplements used to support the parental BL31 cells. As with our previously published single EBNA3 knockout BL31 cell lines, there is no consistent difference in the expression of other EBV genes between the mutant and wild-type-infected cells (Figure S1).

RNA extracted from three or four cell lines for each mutant group (3AKO, 3BKO, 3CKO and E3KO), from two or three lines for each corresponding revertant and the parental EBV-BAC (wtBAC), and three times from the parental BL31 line was used to generate transcriptome information using Affymetrix Human Exon 1.0 ST microarrays, analysed by MMBGX [46] to interpret this at the gene and transcript level.

### EBNA3 genes co-operatively regulate host genes

Principal component analysis and hierarchical clustering both show that the different EBV mutants generally segregate together according to their gene expression profile (Figure S2). The main exception to this is the EBNA3A mutants, two of which cluster with the mutants and two on the edge of the wild-type group. The reason for this heterogeneity is not clear, although it reflects the time at which the cell lines were generated, and the consequence is to reduce the number of genes passing any statistical threshold, reducing the number of genes apparently regulated by EBNA3A. We identified differences in gene expression level by analysis of variance (ANOVA), using contrasts of each mutant (and uninfected BL31) with the wtBAC-infected cell lines (including revertants) (Table S1). False discovery rate (FDR) analysis on the p-values for the contrasts implied that an unadjusted p-value of around 0.001 would offer sufficient statistical confidence. Additionally, there was no statistically significant difference between any revertant group and the rest of the wild-type lines.

**Figure 1:**
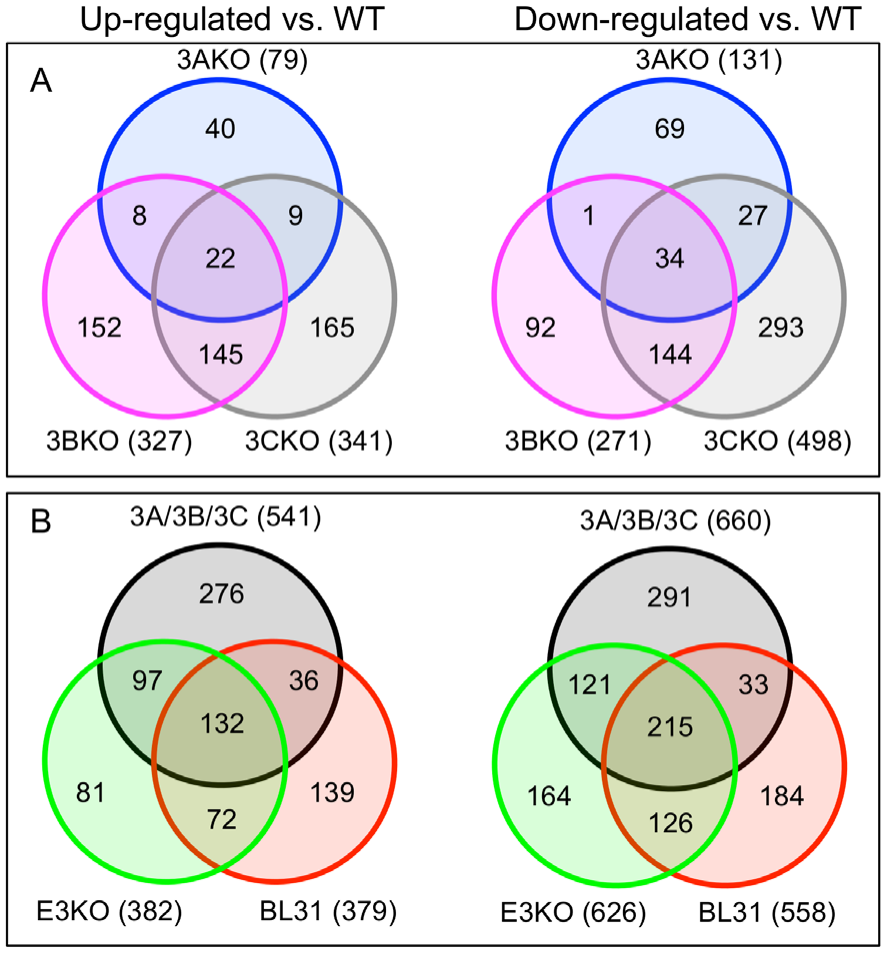
Overlap of genes differentially expressed by EBV mutant BL31 cells. Each set in the Venn diagram contains the number of genes defined as differentially regulated (p value <0.001 and fold change >2) for the mutant group shown as compared to wtBAC-infected BL31. The total number of genes in the set is indicated below the set's identity. **A.** Numbers of genes differentially regulated in different combinations of EBNA3 knockout virus-infected BL31 cell lines are indicated. **B.** The combined group of genes falling in the venn diagrams from A (group 3A/3B/3C) is compared with genes differentially regulated between wtBAC-BL31s and E3KO-BL31s (bottom left set) or uninfected BL31 (bottom right set). Up-regulated vs WT (left side Venn diagrams) indicates genes whose expression was higher in the mutant-infected/uninfected BL31s than wtBAC-infected BL31s; down-regulated vs WT indicates lower expression in these lines.

Using a fold-change of 2 as an arbitrary threshold, along with a p-value of <0.001 we established lists of genes differentially regulated by each of the EBNA3s. Deleting EBNA3C has the greatest impact on the regulation of host genes, with 839 genes affected. In contrast the 3BKO cells showed 598 differentially regulated genes, with 210 differentially regulated in the absence of EBNA3A. For each mutant, the numbers of genes up- and down-regulated are fairly similar (in a ratio of 2∶3 for 3AKO and 3CKO), but a Venn diagram combining the single EBNA3 knockouts shows a large overlap in genes deregulated by deletion of a single EBNA3 gene (Figure 1A). For each set of genes (3AKO, 3BKO or 3CKO) only around half are unique to that set – for instance, 101 of the 210 genes altered in 3AKO BL31 are also significantly altered in at least one of 3BKO or 3CKO BL31 cells. It is striking that EBNA3B, dispensable for the immortalisation of B-cells by EBV, and currently regarded as a minor player in EBV biology, is required for the repression of almost as many genes as EBNA3C, and indeed co-operates with EBNA3C in altering the regulation of about half of these. In contrast, the overlap with EBNA3A in gene repression is much more modest, with few genes solely requiring EBNA3A and 3C (such as *BCL2L11*/*BIM*) or 3A and 3B for their regulation. Indeed where 3A and 3C are involved in gene repression, the norm appears to be that EBNA3B is also required.

In the genes up-regulated by EBV (and thus at reduced levels in the knockouts) a similar pattern of co-operation emerges, although EBNA3C plays a more dominant role. Here, there is less of a role for EBNA3B alone or combined with EBNA3A, and a greater role for the combination of EBNA3A and 3C, and for EBNA3C alone. The inference of this might be that EBNA3C is more of a transcriptional activator than EBNA3B but the steady state nature of this system makes such inferences speculative at best. However, it does suggest that the EBNA3s work as a concerted whole, with 390 of the 1201 differentially regulated genes requiring multiple EBNA3s in this system.

Taking the group of 1201 genes that are differentially regulated between the single knockouts and wild-type cells, we looked at how many were also differentially regulated between either BL31 and wild-type or triple knockout (E3KO) and wild-type (Figure 1B). Around half of the genes whose expression is altered by infection of BL31 by EBV require at least one of the EBNA3s for at least some component of their regulation. Conversely, only around half of the genes identified as differentially regulated in the single EBNA3 knockouts are also seen in either the E3KO or BL31 contrasts. This may be partly a consequence of setting significance thresholds that fail genes due to the smaller size for the E3KO and BL31 groups (3 samples each).

**Figure 2:**
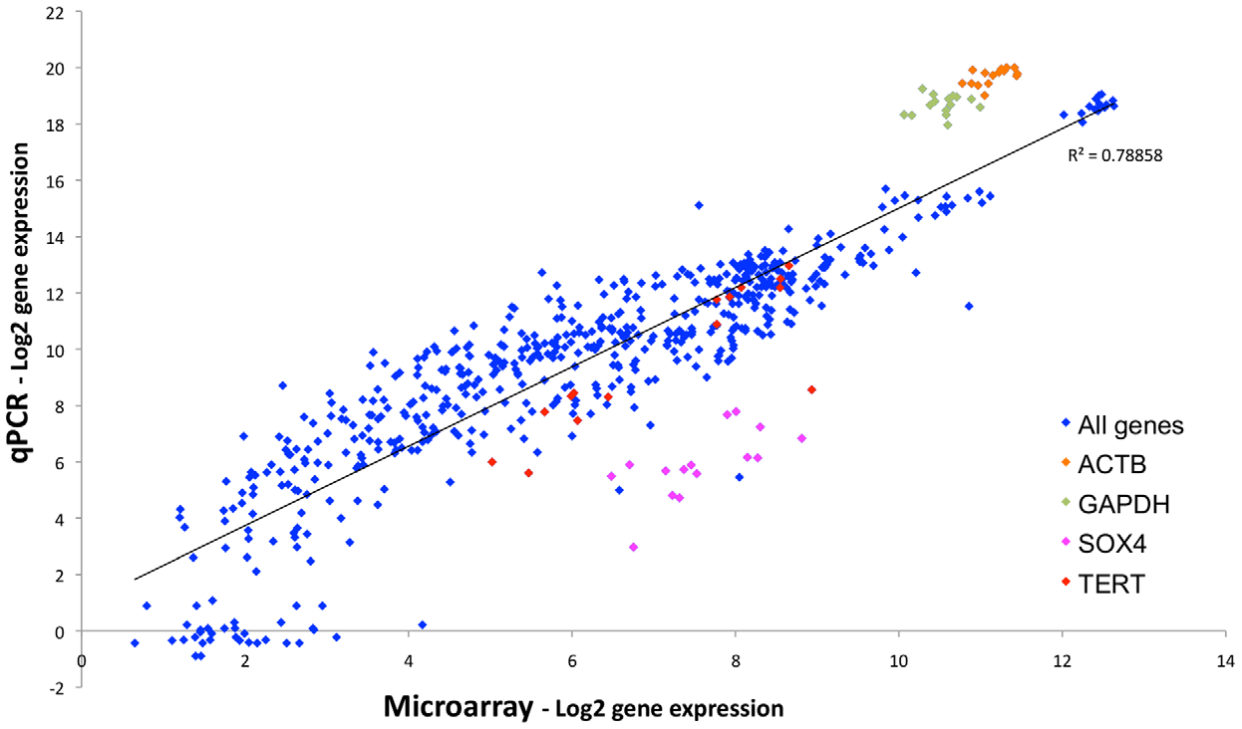
Validation of microarray expression level data by qPCR. The log2-transformed gene expression values of 42 genes were established for 16 of the BL31 cell lines, by qPCR analysis of the RNA samples previously used for the microarrays. These are plotted against the corresponding gene-level expression value from the MMBGX microarray analysis. Correlation coefficient and best-fit line are shown. Assays deviating from this trendline (GAPDH, ACTB and SOX4) are coloured as indicated. TERT is included for comparison with LCL data (Figure S4).

To assess the validity of our microarray data, we undertook PCR analysis of 42 genes, of which 5 were used as endogenous controls to normalise the samples. Plotting the relative quantity as determined by qPCR against the MMBGX gene level estimate (Figure 2), there is generally a good correlation between microarray and qPCR data, although a two-fold change by microarray corresponds to a 2.7-fold change by qPCR. The main deviations from this trend are either: a) where the transcript level fell below the detectable threshold for the qPCR assay, mainly where the microarray log2 expression value <3; b) for the *GAPDH* and *ACTB* endogenous control assays, which appear to be more efficient qPCR assays than the others; and c) the SOX4 qPCR assay, which measured a lower expression level than the microarray but, upon inspection, had an inferior amplification profile (not shown). This suggests that the microarray gene level estimates are a generally accurate reflection of transcript level in the cell population.

### Functional groups enriched in EBNA3-mutant BL31 cells

To explore what classes of genes were regulated by the *EBNA3* genes in BL31 cells, we used the online ontology resource, DAVID. In order to avoid missing biological relationships because of the arbitrary nature of the cut-offs used to generate gene lists, we compiled additional lists of up and down-regulated genes for each of the EBNA3 knockouts, using more stringent p-value criteria (<0.0001) for smaller fold-changes (>1.3). The most striking observation was the enrichment of genes up-regulated in the 3CKO BL31 cells for the GOTERM-BP ‘cell cycle’ with a q-value significance of 6.8×10^−25^. Highly significant enrichments were also seen for ‘mitosis’ and ‘M-Phase’, ‘chromosome organisation’ (mainly histone clusters) and ‘cytoskeleton’, suggesting that this alteration is related to mitosis. Curiously, where the fold-change threshold is increased to 2, no significant enrichment of these ontological groups is observed. Enrichment of these categories is also seen for 3BKO up-regulated genes, but only for the subset co-regulated by EBNA3C.

**Figure 3:**
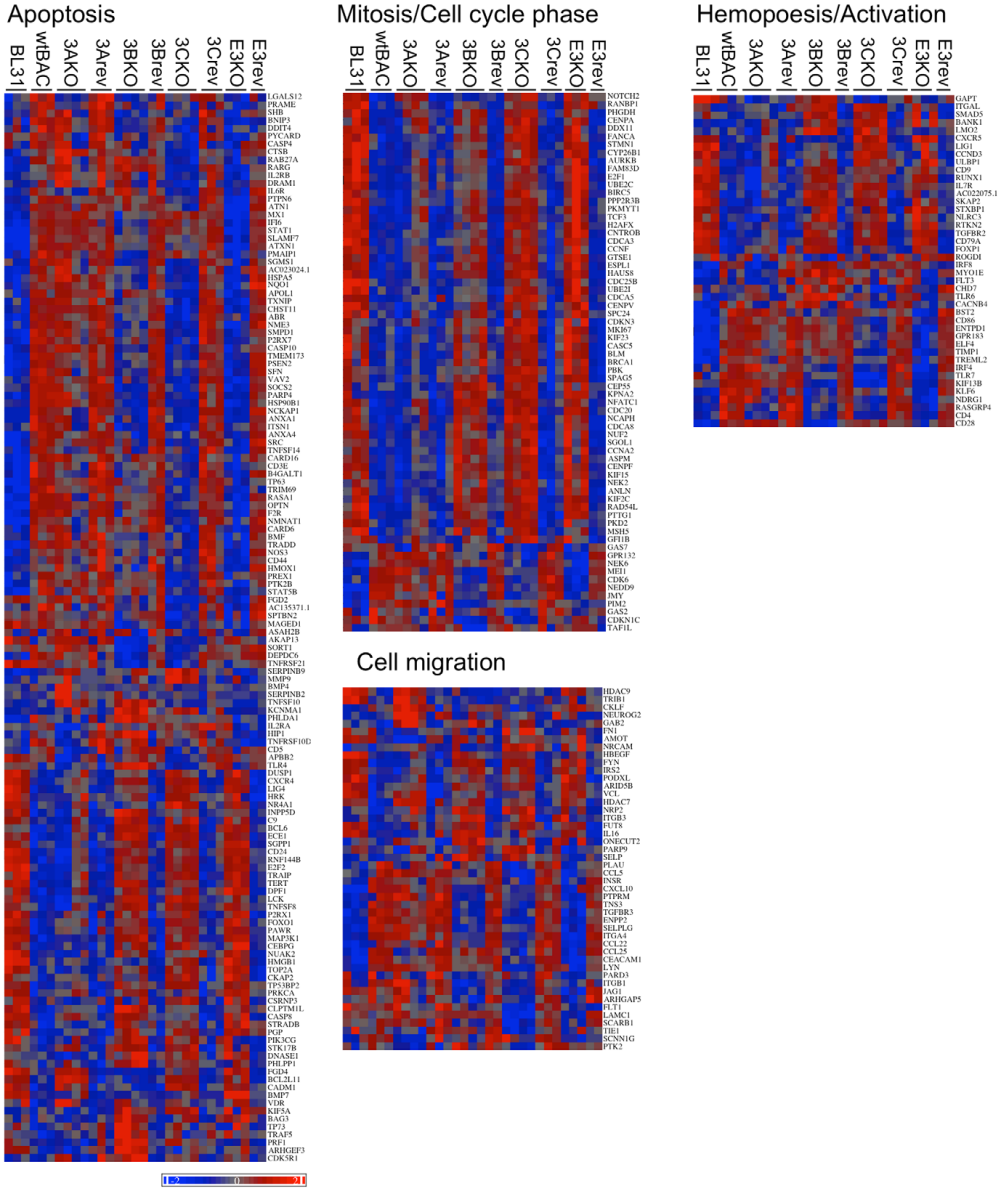
Relative expression of differentially regulated genes falling into significant ontological groups. MMBGX-extracted gene expression levels were normalised to the mean and scaled by the standard deviation of the gene expression level for each gene – colour coding ranges from +2 to −2 standard deviations (see scale bottom left). These were grouped by ontological term, and where genes fell into multiple groups, they were placed in the group in the following order – Mitosis, apoptosis, cell migration, hemopoesis and activation. Genes within each group were ordered by unsupervised clustering.

Functional clustering analysis indicated additional biological processes that may be altered by the deletion of the EBNA3 genes. These are involved with the processes of cell migration/locomotion/chemotaxis, lymphocyte activation and differentiation, and apoptosis and its regulation – the statistics for enrichment of these clusters for the various mutant groups (with up-and down-regulated genes for each mutant treated both as separate gene sets and as a single group) are detailed in Table S2. Viewing the expression profiles of the genes in these clusters (Figure 3) the co-operative nature of the EBNA3s in manipulating host gene expression is evident. Taken overall, the large number of genes co-regulated by EBNAs 3B and 3C is clear, while individual genes regulated by other combinations can be easily picked out. For instance, *BIM* (*BCL2L11* - towards bottom of apoptosis heat map) whose expression in 3AKO and 3CKO BL31 cells correlates with susceptibility to apoptotic stimuli is expressed similarly to CADM1 and FGD4.

### EBNA3 deletion alters expression levels of genes involved in B-cell differentiation

**Figure 4:**
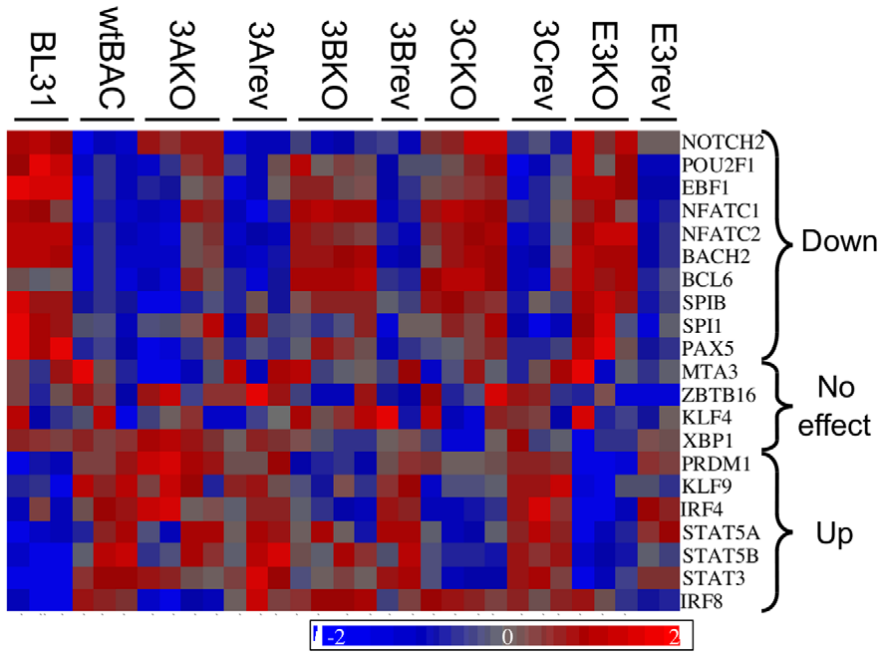
EBV and EBNA3s alter the expression of B-cell differentiation-related transcription factors. Heat map shows the relative expression levels of key transcription factors involved in B-cell differentiation. MMBGX-extracted gene expression levels were normalised to the mean and scaled by the standard deviation of the gene expression level for each gene – colour scale ranges from +2 to −2 standard deviations. Genes are grouped according to whether they are up- or down-regulated upon infection by EBV. Those not significantly altered are also indicated. Fold change and ANOVA contrast p values for these genes are shown in Figure S2.

Since different patterns of EBV gene expression are known to be coupled to the differentiation state of the host B cell, we examined the gene expression profiles of 21 transcription factors associated with the B cell differentiation process [47], [48], [49]). Of these, 17 were altered at the transcript level by EBV, and many required one or more of the EBNA3s for this regulation (Figure 4). Notably, *BCL6*, *NFATC2* and *BACH2* exhibit more than a 10-fold increase in RNA levels in one or more mutant and EBV infection also alters *EBF1* and *STAT5A* more than five-fold (Table S3).

Additionally, we performed gene set enrichment analysis (GSEA) for EBV- and EBNA3-regulated genes among those whose expression changes through the germinal centre transition [50]. This suggested that EBV-infection of the BL31 cell alters its phenotype away from that of a germinal centre cell, and that EBNA3C is required for the modulation of some of these genes. EBNA3B deletion results in the up-regulation of a subset of genes that are normally more repressed in the germinal centre, but does not significantly affect genes up-regulated in the germinal centre (see Supporting Information File S1 and Table S4 for full description).

### Epigenetic promoter modifications reflect expression changes

**Figure 5:**
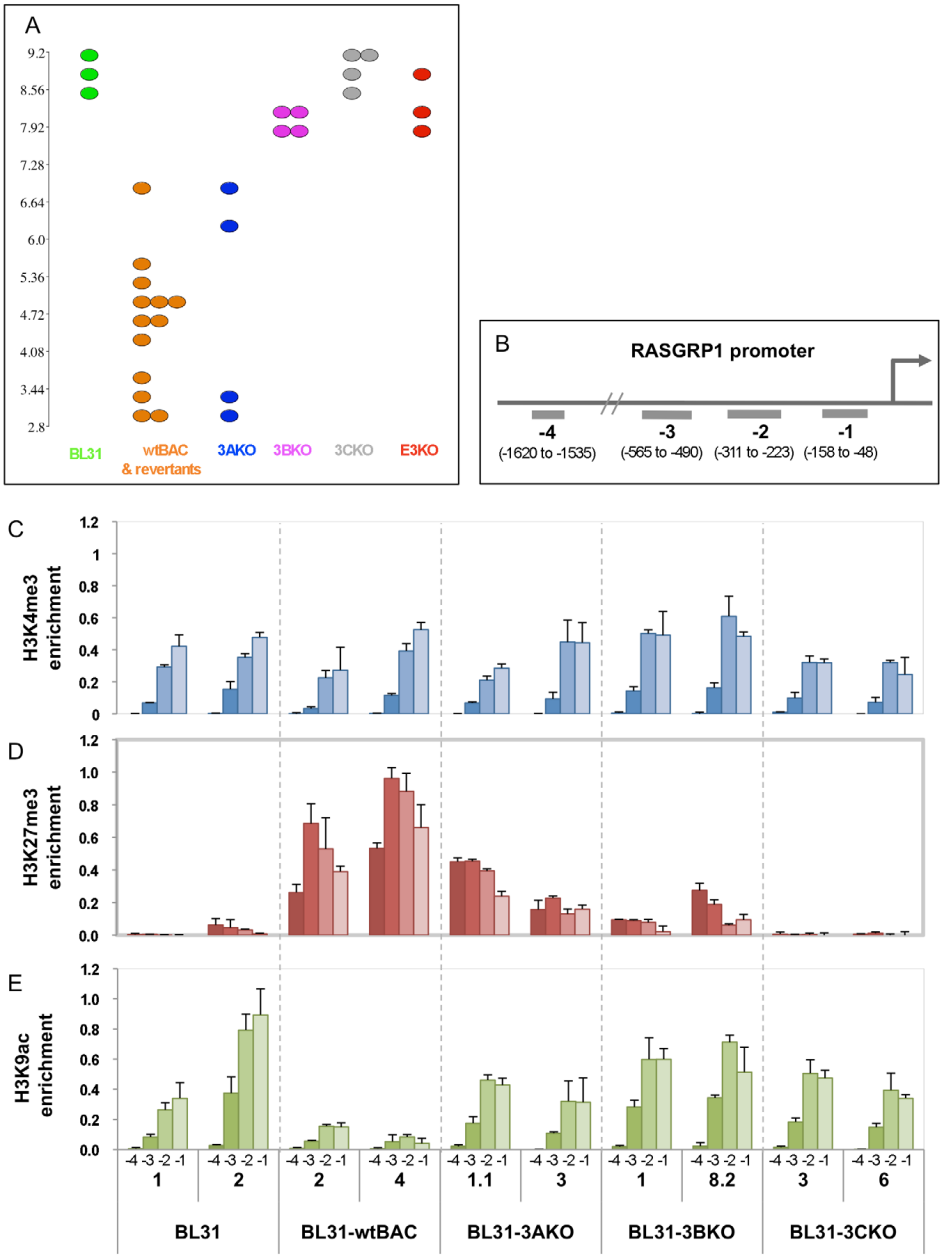
Expression and epigenetic marks associated with a gene regulated by EBNA3B and EBNA3C; RASGRP1. (**A**) Dotplot visualisation of expression level values of individual BL31 cell lines. EBV mutant used to generate the cell line is indicated below and colour-coded (BL31 indicates EBV-negative BL31 cell lines). Y axis shows expression level (MMBGX gene level analysis) on a log2 scale (ie each unit change indicates a doubling). (**B**) Schematic representation showing the location of ChIP qPCR assays relative to the transcriptional start site of the RASGRP1 promoter. (**C-E**) Bar charts indicate the percentage of input DNA recovered for each assay and each cell line for ChIPs using antibodies against trimethylated H3K4 (**C**), trimethylated H3K27 (**D**), and acetylated H3K9 (**E**). Numbers −1 to −4 relate to the assay position. Numbers below these are the clone IDs of the cell lines used.

**Figure 6 pone-0013979-g006:**
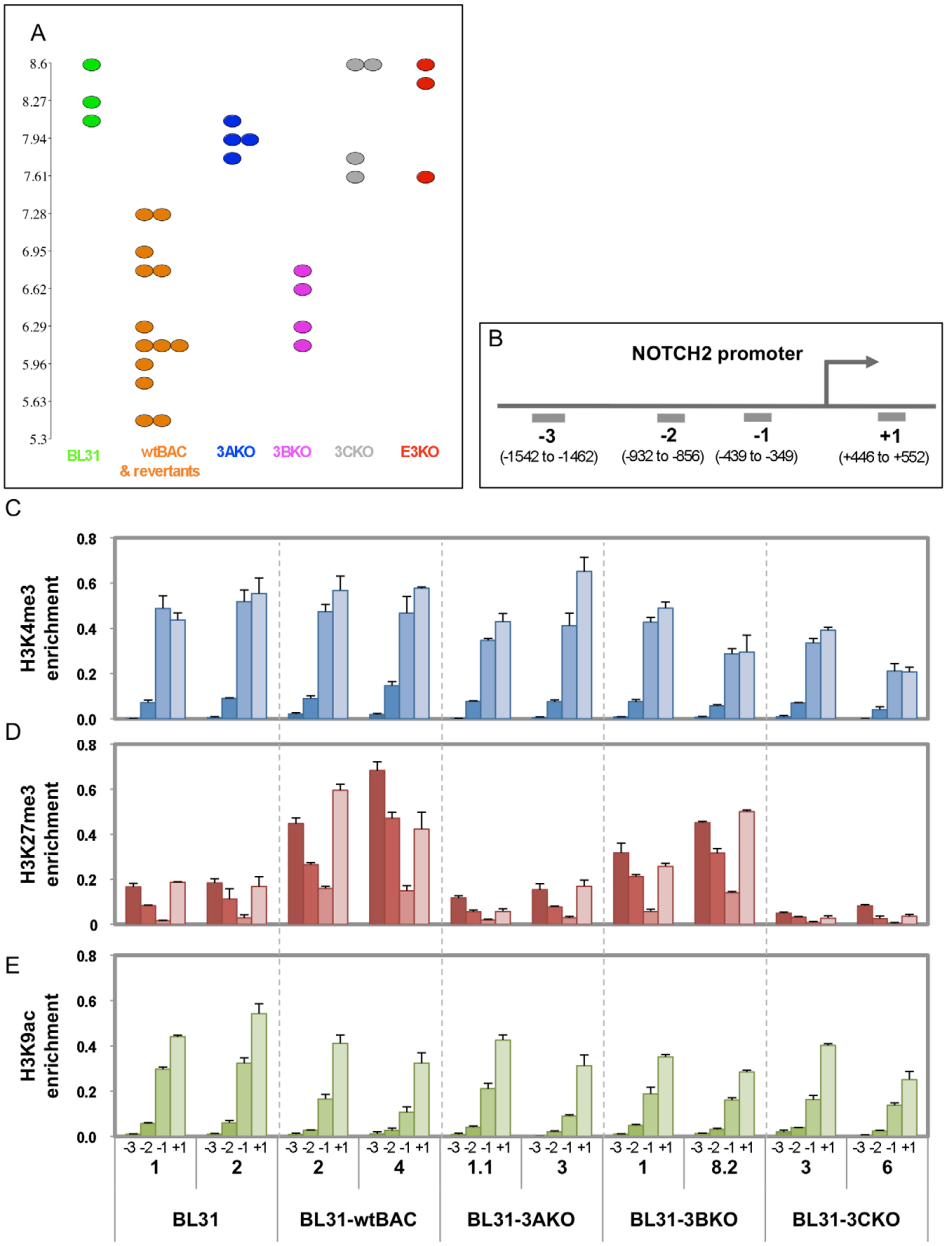
Expression and epigenetic marks associated with a gene regulated by EBNA3A and EBNA3C; NOTCH2. (**A**). Dotplot visualisation of expression level values of individual BL31 cell lines as described for figure 5A (**B**). Schematic representation indicates of location of ChIP qPCR assays relative to the transcriptional start site of the NOTCH2 promoter. (**C-E**) Bar charts indicate the percentage of input DNA recovered for each assay and each cell line, for ChIPs using antibodies against trimethylated H3K4 (**C**), trimethylated H3K27 (**D**), and acetylated H3K9 (**E**). Numbers +1 to −3 relate to the assay position. Numbers below these are the clone IDs of the cell lines used.

**Figure 7 pone-0013979-g007:**
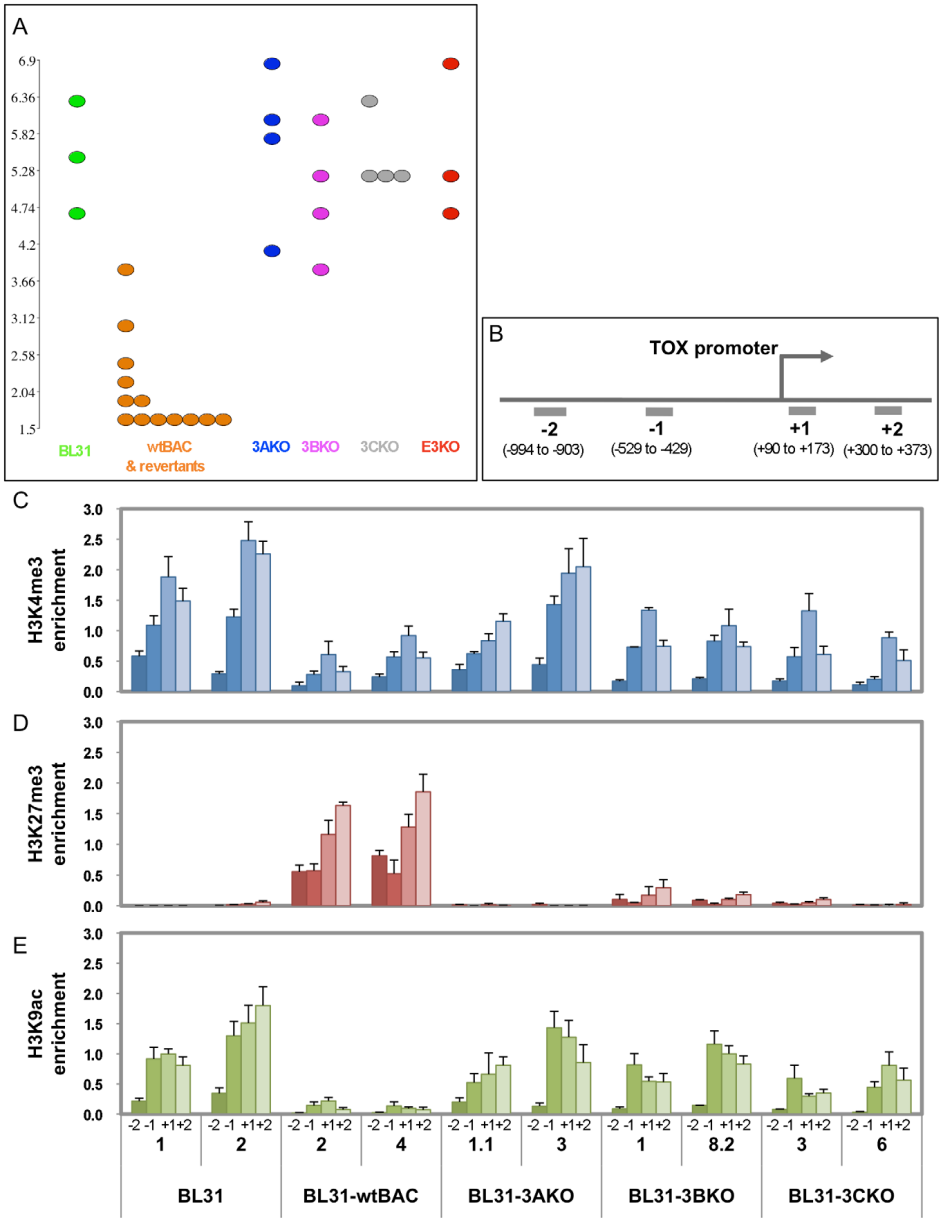
Expression and epigenetic marks associated with a gene regulated by EBNA3A, EBNA3B and EBNA3C; TOX. (**A**) Dotplot visualisation of expression level values of individual BL31 cell lines as described for figure 5A (**B**). Schematic representation indicates of location of chromatin immunoprecipitation primer assays relative to the transcriptional start site of TOX promoter. (**C-E**) Bar charts indicate the percentage of input DNA recovered for each assay and each cell line, for ChIPs using antibodies against trimethylated H3K4 (**C**), trimethylated H3K27 (**D**), and acetylated H3K9 (**E**). Numbers +2 to −2 relate to the assay position. Numbers below these are the clone IDs of the cell lines used.

In order to further validate the observations from our microarray we selected individual genes that represented the different co-regulated groups for the microarray. Since the designation of a gene to any group is based on partially arbitrary cut-offs, we selected genes that convincingly fell into each group, having the same change in expression level for each mutant, as well as clearly exhibiting no change for the other mutant group. We immunoprecipitated chromatin modified by trimethylation of lysines 4 and 27 and acetylation of lysine 9 of histone H3 (H3K4Me3, H3K27Me3 and H3K9Ac respectively), and used qPCR directed at regions around the gene promoter to identify the chromatin modifications at each promoter. The genes used were *NOTCH2* for EBNA3A/3C co-regulated (Figure 5); *RASGRP1* for 3B/3C (Figure 6) and *TOX* for 3A/3B/3C (Figure 7). Each figure shows the microarray data (A) confirmation of gene expression by qPCR (B) and the ChIP data (C) with the position of the ChIP qPCR assays indicated below.

For *NOTCH2*, *RASGRP1* and *TOX*, we can clearly see an inverse correlation between expression and trimethylation of H3K27. For instance, the derepression of *NOTCH2* in 3AKO and 3CKO-BL31 cell lines is accompanied by a corresponding reduction in H3K27 trimethylation. Similarly, *RASGRP1* has less H3K27Me3 in 3BKO and 3CKO BL31, and *TOX* only has appreciable levels of H3K27Me3 in the wtBAC-BL31 cell lines. In contrast, H3K4 trimethylation of the promoters, generally a mark of transcriptionally active genes, is largely unchanged between the cell lines, while H3K9 acetylation correlates with increased expression of *TOX* and *RASGRP1*, but is unchanged on the *NOTCH2* promoter. The significance of these epigenetic combinations is considered below.

### A subset of genes is differentially expressed in both 3BKO-LCLs and 3BKO BL31 cell lines

In order to see to what extent our observations transfer beyond the BL31 cell line, we investigated the effect of EBNA3B deletion in LCLs, as EBNA3B is known to be dispensable for B cell immortalisation [12]. Using the 3BKO and wtBAC viruses, we established two independent LCLs in lymphocytes from each of three independent EBV-negative blood donors. The 3BKO-LCLs established efficiently, perhaps more readily than wtBAC LCLs, and analysis of EBV latent protein levels showed that other than the loss of EBNA3B expression, EBV proteins did not differ substantially between wtBAC-LCL and 3BKO-LCLs (Figure S3). RNA harvested from these lines between 3–6 weeks after infection was analysed on Affymetrix Human Exon 1.0 ST microarrays. A 2-way ANOVA model incorporating the donor ID (representing the three different genetic backgrounds of the transformed cells) as the additional factor revealed considerable gene expression changes between wtBAC and 3BKO LCLs (Table S5). A p-value of 0.0004-7 corresponded to a 5% FDR but, for a more representative comparison with the BL31s, we have used a p-value of 0.001 as a statistical threshold in our analysis.

Analysis of gene expression levels by qPCR again showed a high degree of correlation with gene expression values from the microarray (Figure S4), with the same outlying assays (*ACTB*, *SOX4* and *GAPDH*) as seen for the BL31 analysis. Additionally, *TERT* mRNA was often undetectable by qPCR in the wtBAC LCLs, while the microarray implies a modest down-regulation. Since this observation was true for five independent TERT qPCR assays spanning different exon junctions (Figure S5 A-D), it suggests that the microarray signal for this gene is artificially high in these cells, or that the amplification of the transcript is somehow prevented.

**Figure 8:**
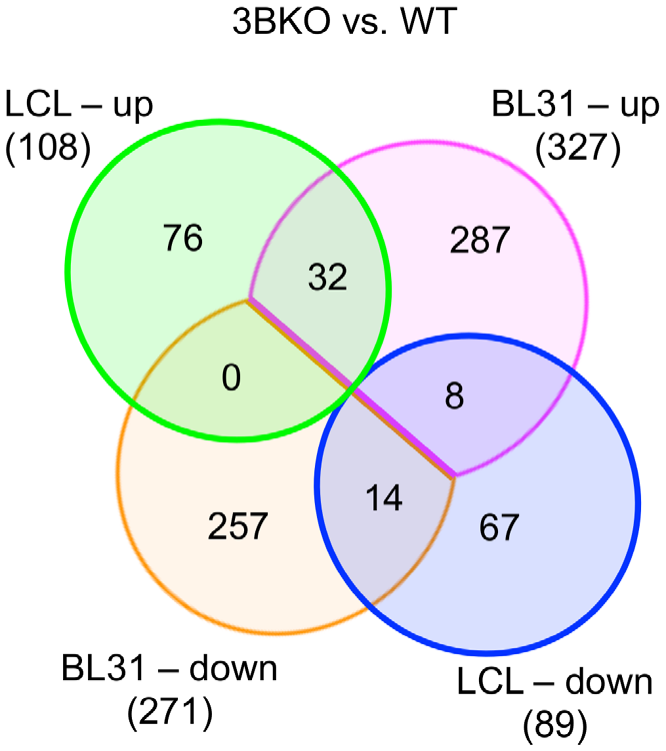
Overlap in EBNA3B-regulated genes between LCL and BL31 cell lines. Venn diagram showing overlaps of genes whose regulation changes more than two-fold (p<0.001). The top two sets are of genes more highly expressed in the EBNA3B knockout cell lines than wtBAC cells; bottom two are down-regulated in 3BKO lines as labelled. The identities of the genes differentially expressed in both 3BKO BL31 and LCLs, and their ANOVA statistics are given in Table S6.

Comparing the genes regulated by EBNA3B in LCLs with those of BL31, we see only a modest overlap (Figure 8; Table S6). Of the 89 genes requiring EBNA3B for their repression in LCLs, 14 are also repressed in BL31s, while of the 108 genes up-regulated in 3BKO LCLs, 32 are also up-regulated in BL31-3BKO. A further eight of the genes that are down-regulated in 3BKO LCLs are up-regulated in BL31s. Of these eight genes, four (*ARID5B*, *CD72*, *RUNX1*, *TNFSF10*) are also significantly altered during the naïve to centroblast transition of the germinal centre reaction, and a further 3 are not uniquely represented on the U133 microarray used in that experiment [50]. Taken together, this shows that some 25% of EBNA3B-regulated genes in LCLs also require EBNA3B for their regulation in BL31.

**Figure 9:**
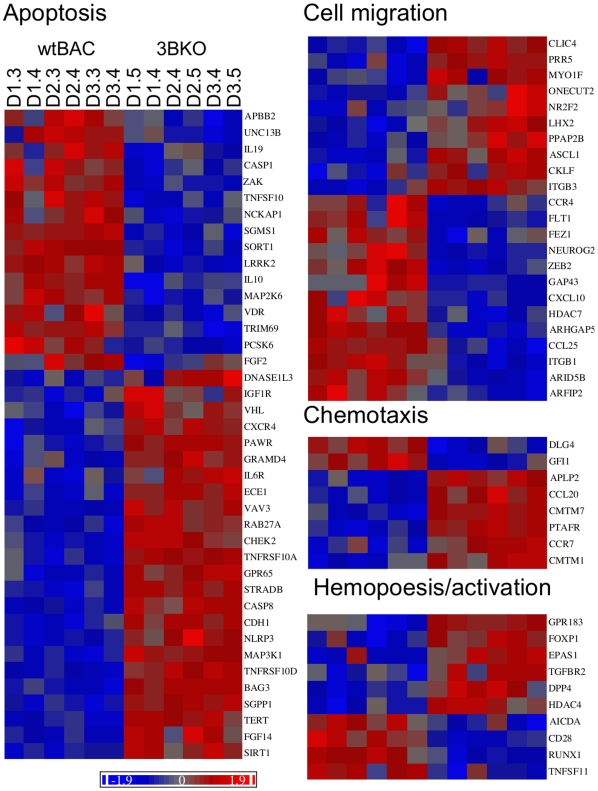
Ontological groupings of EBNA3B-regulated genes in LCLs. MMBGX-extracted gene expression levels were normalised to the mean and scaled by the standard deviation of gene expression levels in LCLs for each gene, and coloured by relative expression (see scale). These were grouped by ontological term, and where genes fell into multiple groups, they were placed in the group in the following order - apoptosis, cell migration, chemotaxis and then hemopoesis/activation. Genes within each group were ordered by unsupervised clustering. Cell clone IDs are indicated at the top of the apoptosis heatmap and are in the same sequence for other categories.

In common with the analysis of gene ontology enrichment in 3BKO BL31, genes involved with the regulation of apoptosis were among the most significantly altered in 3BKO LCLs. Additionally genes involved with cell migration and its regulation, and membrane expression (signal-anchor, glycoprotein, membrane and Golgi localisation) were also enriched, with 43.8% of the regulated genes being tagged by the keyword ‘membrane’ in Uniprot annotation (q-value = 0.000068), suggesting that EBNA3B LCLs may exhibit a distinct cell surface phenotype from wtBAC LCLs (Figure 9 and Table S7). On the whole, the enrichment scores are modest, and using separate up- and down-regulated gene lists does not enhance the enrichments.

### EBNA3A and EBNA3B co-operate in gene regulation in LCLs

A recent study of LCLs generated with mutants of EBNA3A in the same B95-8 EBV-BAC background identified nearly 300 genes whose expression differed from wild-type LCLs. This study used two EBNA3A mutants [lacking either the whole gene (3AmutB) or just the second exon (3AmutA)] which differed somewhat in the genes they regulated (not shown). For a rational comparison with our cell lines, which are complete EBNA3A knockouts, we have identified the genes differentially regulated between 3AmutB and WT LCLs from the published microarray data.

**Figure 10:**
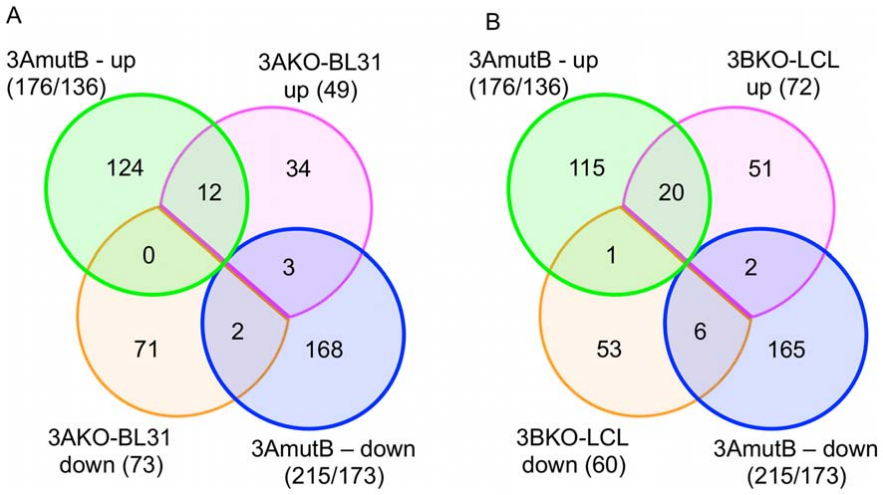
Consistency between 3AKO BL31 and LCLs, and co-operation between EBNA3B and EBNA3A in LCLs. Venn diagrams indicate overlap between genes differentially regulated [i.e. with a minimum fold change of 2 and p<0.001] in (**A**) 3AmutB LCLs and 3AKO BL31; and (**B**) 3AmutB and 3BKO LCLs. Numbers of genes are indicated in the sets and the size of each set indicated below the set name. For 3AmutB this is denoted as “(no of probesets/no of unique gene IDs)”. The 3AKO-BL31 and 3BKO-LCL sets exclude genes not represented in the U133 Plus2 microarray that was used to generate the 3AmutB LCL data.

Comparing these genes (having filtered our array data to include only genes represented in the U133 microarray that was used for the mutB LCLs), we see a modest overlap (23% of the BL31 group) in genes derepressed by EBNA3A deletion in both cell types but very little overlap for genes whose expression falls in 3AKO LCLs (Figure 10A). These genes include the aforementioned *NOTCH2* as well as *PRKCA*, *HDAC9*, *TNFRSF10* (*TRAIL*), *BMP4* and the vitamin A transporter *SLC23A2* (Table S7).

The overlap between the EBNA3A mutant and 3BKO LCLs is substantial for genes whose expression rose in the mutants, with 20 of 72 (around 25%) of the 3B-repressed genes also being derepressed in EBNA3A mutant LCLs for at least one probeset (Figure 10). Expressed as a proportion of 3A-regulated genes (20 of 136) this proportion is considerably smaller, but unlike 3BKO-LCLs, the EBNA3A mutant LCLs have impaired growth rates and increased levels of pro-apoptotic factors [10], [11], so many of the additional differences could be due to alterations in gene expression associated with these changes.

The proportion of genes up-regulated in both mutants is smaller, only six of 60 (10%), with a further three genes being up in one line and down in the other. All these genes, (plus three which marginally fail the significance test for mutB but are significant when all the 3A mutants are treated as a single group) are summarised in Table 1. Looking at the expression data for these genes in BL31 cell lines provides additional indicators as to their regulation.

**Table 1:** Genes whose expression is regulated by EBNA3B and EBNA3A in LCLs.

		3BKO vs wtBAC		U133 probeset ID	3AmutB vs WT		BL31 Changes
Ensembl Gene ID	Gene Name	p-value	Fold-Change		p-value	Fold-Change	
ENSG00000224220	AC104699.2	**0.0008284**	**−2.01**	**215565_at**	**0.0000363**	**−4.76**	EBV activates; no EBNA3 effect
ENSG00000102760	C13orf15	**0.0000037**	**−4.69**	**218723_s_at**	**0.0000522**	**−4.20**	None
ENSG00000064763	FAR2	**0.0000123**	**−9.05**	**220615_s_at**	**0.0001155**	**−5.13**	Not significant
*ENSG00000089692*	*LAG3*	***0.0004815***	***−3.63***	***206486_at***	*0.0042367*	***−3.12***	*None*
ENSG00000070759	TESK2	**0.0008605**	**−2.18**	**205486_at**	**0.0000122**	**−2.91**	3AKO, 3BKO and 3CKO down <2-fold
ENSG00000181458	TMEM45A	**0.0000045**	**−2.10**	**219410_at**	**0.0000083**	**−10.53**	None
ENSG00000121858	TNFSF10	**0.0002546**	**−2.28**	**202687_s_at**	**0.0001065**	**−3.51**	3AKo and 3BKO up
				**202688_at**	**0.0001249**	**−8.98**	
				214329_x_at	0.0014427	**−2.03**	
ENSG00000197381	ADARB1	**0.0005489**	**6.49**	**203865_s_at**	**0.0000001**	**7.45**	3BKO up 6x
				207999_s_at	0.2306420	1.14	
				209979_at	0.5874880	1.06	
ENSG00000084234	APLP2	**0.0000116**	**2.82**	**208248_x_at**	**0.0000074**	**2.96**	None
				**208702_x_at**	**0.0002423**	**2.08**	
				**214875_x_at**	**0.0000891**	**2.15**	
				**211404_s_at**	**0.0000544**	**2.50**	
				**208704_x_at**	**0.0001191**	**2.58**	
				**208703_s_at**	**0.0007441**	**3.05**	
ENSG00000139352	ASCL1	**0.0000428**	**26.68**	**213768_s_at**	**0.0001322**	**3.99**	None
				209985_s_at	**0.0006246**	1.51	
				**209987_s_at**	**0.0009894**	**6.68**	
				209988_s_at	0.0064834	**6.19**	
ENSG00000151929	BAG3	**0.0000045**	**8.60**	**217911_s_at**	**0.0000022**	**5.62**	3BKO up
ENSG00000064012	CASP8	**0.0000325**	**2.29**	**213373_s_at**	**0.0000005**	**4.54**	3AKO, 3BKO and 3CKO up 1.5-1.8x
				**207686_s_at**	**0.0003986**	**3.89**	
ENSG00000115009	CCL20	**0.0001118**	**4.61**	**205476_at**	**0.0005208**	**17.27**	None
ENSG00000081377	CDC14B	**0.0000240**	**5.09**	**221556_at**	**0.0000014**	**10.98**	None
				**208022_s_at**	**0.0000405**	**5.16**	
				**221555_x_at**	**0.0001045**	**3.50**	
				211348_s_at	0.0135807	1.21	
				211347_at	0.0496798	1.16	
				216284_at	0.4095960	1.06	
ENSG00000039068	CDH1	**0.0000552**	**6.85**	**201131_s_at**	**0.0001482**	**9.01**	3AKO, 3BKO and 3CKO down 2x (marginal significance)
				201130_s_at	0.0137153	**2.22**	
ENSG00000146592	CREB5	**0.0000003**	**11.14**	**205931_s_at**	**0.0000181**	**5.18**	3BKO up
ENSG00000197872	FAM49A	**0.0000665**	**12.68**	**208092_s_at**	**0.0000001**	**16.97**	3AKO up
				**209683_at**	**0.0000001**	**14.07**	
ENSG00000132589	FLOT2	**0.0000025**	**3.83**	**201350_at**	**0.0002471**	**3.17**	3B 3C 1.3-1.6x
				**211299_s_at**	**0.0002721**	**3.26**	
ENSG00000152804	HHEX	**0.0000072**	**19.30**	**215933_s_at**	**0.0000263**	**6.60**	3BKO and 2 of 4 3AKO up
				204689_at	0.0011165	**3.73**	
ENSG00000198825	INPP5F	**0.0000005**	**23.56**	**203607_at**	**0.0000003**	**11.10**	3BKO up 20x; 3CKO up 2x
ENSG00000239961	LILRA4	**0.0003930**	**5.15**	**210313_at**	**0.0000011**	**6.00**	3BKO up
ENSG00000049759	NEDD4L	**0.0000795**	**2.84**	**212448_at**	**0.0000537**	**2.06**	E3KO and BL31 down 3.5x
				212445_s_at	0.0014747	**2.81**	
ENSG00000100784	RPS6KA5	**0.0003159**	**3.16**	**204633_s_at**	**0.0007989**	**2.00**	None
				204635_at	0.0597242	1.42	
ENSG00000089057	SLC23A2	**0.0000042**	**8.46**	**209237_s_at**	**0.0000344**	**3.08**	3BKO up 5x; 3AKO and 3CKO up 3x
				**211572_s_at**	**0.0001151**	**3.85**	
				**209236_at**	**0.0000987**	**3.42**	
*ENSG00000088992*	*TESC*	***0.0001774***	***3.12***	***218872_at***	*0.0017321*	***4.27***	*3CKO down 5x*
ENSG00000111962	UST	**0.0003600**	**4.55**	**205138_s_at**	**0.0000059**	**3.25**	EBV infection up 10x; 3BKO up 2x more and 3AKO down (not significant)
				**205139_s_at**	**0.0000124**	**9.74**	
ENSG00000047644	WWC3	**0.0000148**	**3.92**	**219520_s_at**	**0.0004171**	**2.45**	3BKO and 3CKO up
ENSG00000093100	XXbac-B461K10.1	**0.0000028**	**7.15**	**212715_s_at**	**0.0000550**	**2.80**	3BKO up
							
ENSG00000169245	CXCL10	**0.0009958**	**−7.89**	**204533_at**	**0.0000000**	**37.04**	3BKO 3CKO and 2 of 3AKO down >4x
*ENSG00000185432*	*METTL7A*	***0.0000215***	**−** ***3.50***	*207761_s_at*	*0.0023597*	***6.08***	*3BKO down*
				209703_x_at	0.5304590	1.06	
							
ENSG00000115525	ST3GAL5	**0.0000387**	**6.68**	**203217_s_at**	**0.0001469**	**−2.48**	3AKO down 3BKO up marginal significance
ENSG00000169403	PTAFR	**0.0000137**	**8.45**	**211661_x_at**	**0.0004363**	**−2.83**	3BKO up 1.4x; 3AKO down 1.7x
				206278_at	0.0361156	−1.27	

Genes are grouped into those down-regulated in EBNA3A and 3B knockout LCLs (top part), up-regulated in both (middle), and regulated in different directions in the two mutants (Bottom). For each gene, data for all of the Affymetrix U133plus2 probesets mapped to that gene are included. ANOVA p-values and fold changes are shown for 3BKO-LCL vs wtBAC LCL and for 3AmutB LCL vs WT LCL. Affymetrix IDs are **bold** where p<0.001 and fold change >1.5. Data are **bold** where p<0.001 or fold change >2.

*Genes in italics passed significance thresholds for all EBAN3A mutants vs WT, but not quite for 3AmutB vs WT.* The changes seen in the gene's expression in BL31 cells are indicated in the final column.

## Discussion

Our analysis of EBNA3 function in regulating host gene expression has been undertaken in two cell backgrounds. BL31 is a Burkitt's lymphoma-derived post-germinal centre B-cell line. In contrast, LCLs are derived from the infection of naïve resting B-cells, relying on the functions of EBV to transform and immortalise the infected cell. These two backgrounds offer different advantages and drawbacks that should be borne in mind when considering the implications of the microarray data. In BL31, EBV is operating in a background where various signalling and transcriptional pathways are likely to have been distorted during oncogenesis and cell line outgrowth, most notably the constitutive activation of the c-Myc oncogene characteristic of BL. However, functions of EBV are not required for the proliferation of these cells, which allows the analysis of any EBV mutant capable of generating infectious particles and persisting episomally. In contrast, LCLs are much closer to the physiological situation of infected B cells. However, the reliance on EBV's immortalizing ability prevents study of 3CKO and E3KO mutant viruses, as LCLs could not be established with these viruses (data not shown). Additionally, the impaired outgrowth and proliferation of 3AKO LCLs complicates the analysis [10], [11], in contrast to the robust growth of 3BKO and wtBAC LCLs. Culture-specific selection pressures also distort the expression profile of LCLs as they adapt to culture conditions [51]. Additionally, mutant-specific selection pressures can differ from wild-type, as cells compensate for missing transforming functions (see for example [11]). Indeed, any stable cell system can suffer from this issue.

The chief alternative to stable cell lines is to study conditional, inducible or knockout systems. While some of these have been usefully applied to gene expression studies of EBNA2 (albeit with considerable differences in the genes identified by the studies) [52], [53], [54], the conditional EBNA3C is very slow to switch off, is then degraded, and may not retain completely wild-type function [11], [55]. This complicates experimental design and interpretation. Additionally, the EBNA3s are associated with establishing epigenetic repression [26], so in trying to identify key transcriptional targets it is important to vary EBNA3 expression during the imposition of these epigenetic modifications (which presumably occurs early during infection).

It is with these caveats in mind that we consider our findings. Technically, the microarray gene level expression estimates made by MMBGX appear robust and, if anything, highlight inadequacies in the qPCR assays used to validate them (Figures 2 and S4), with the failure to detect hTERT mRNA in wtBAC-LCLs the only exception. We also generated expression data at the transcript level, but the large numbers of annotated transcripts, many of which appear to be partial, appears to have compromised this approach. Analysis of potential alternate transcripts by qPCR suggests that these data can offer an indication of which transcript isoforms are used, but is not a reliable measure of the relative magnitude of these effects (see Supporting Information File S1 and Figure S5).

To support this refinement, and allow our data to be interrogated on a gene-by-gene basis, at both the gene and transcript level, we have generated dotplots of the raw MMBGX output data (as seen in Figures 5A, 6A and 7A) for each gene/transcript and made them available at www.epstein-barrvirus.org.uk. Here, users can type the name of their gene of interest to visualise the gene- and transcript-level expression data for the BL31 and LCL cell lines analysed in our lab, and of the U133 microarray data from the EBNA3A mutant-LCLs produced in the Kempkes lab [10].

Reassuringly, the transcriptome data from the wild-type and revertant cell lines and the EBNA3B and EBNA3C and newly generated E3KO mutants clustered by their virus type but the two pairs of 3AKO cell lines appear rather distinct (Figure S2). Two of these lines (BL31-3AKO-1.1 and -1.2) were generated in unsupplemented media by a low titre virus stock, and took over two months to establish. This combination may have resulted in a powerful selection pressure that has prevented the manifestation of some changes associated with the deletion of EBNA3A in the presence of supplemented medium. This phenomenon can be seen in the dotplot for *RASGRP1* (Figure 5A) in which the two older cell lines express very low levels of the transcript, whereas the other two (BL31-3AKO-3 and -4) express higher transcript levels. This transcriptional difference is also reflected in the H3K27 trimethylation of the locus, with 3AKO-3 exhibiting lower levels than 3AKO-1.1 (Figure 5D). For most loci (e.g. *TOX*, *NOTCH2* - Figures 6 and 7) the difference does not occur, but we have not identified any alteration in EBV gene expression, or any other cause of this anomaly, just as the mechanism for the gene expression differences between 3AmutA and 3AmutB remains unclear [10].

We have previously shown the requirement for both EBNA3A and EBNA3C in the repression of the pro-apoptotic BH3 protein BIM by EBV [26], [42] and of p16^INK4A^ expression in LCLs [11]. Here we find that this requirement for multiple EBNA3 proteins for gene regulation is extremely common (as visualised in figure 3) - around half of the genes whose expression is altered by deletion of each EBNA3 gene also being altered by deletion of at least one other (Figure 1). Direct interaction between EBNA3s in mediating these effects seems likely since, when over-expressed, the EBNA3s all partially co-localise with each other by immunofluorescence [56], [57], while a yeast-2-hybrid screen identified EBNA3A and EBNA3C as interaction partners [58].

The repression of both the *BIM* (*BCL2L11A*) and *p16^INK4A^* (*CDKN2A)* genes by EBNA3A and EBNA3C is marked by H3K27 trimethylation [11], [26], an epigenetic modification mediated by the polycomb complex PRC-2 [27], [59]. To extend our analysis of the epigenetic state of EBNA3-regulated genes, we selected one gene repressed by each co-operating group, where the mean fold change in expression for each mutant was about the same (3B+3C, 3A+3C and 3A+3B+3C, represented by *RASGRP1*, *NOTCH2* and *TOX* respectively). We observed a good correlation between gene repression and the repressive mark H3K27 trimethylation (Figures 5D, 6D and 7D), suggesting that EBNA3B, as well as EBNAs 3A and 3C, is involved in repression through polycomb recruitment. Although EBNA3C is required for the repression of the genes for which ChIP data are presented here, it is not a requirement for EBV-related repression *per se* - loss of repression of *INPP5F* in 3BKO BL31 cells is also characterised by reduced H3K27 trimethylation (data not shown).

In contrast, one mark of activation, H3K4 trimethylation, is present but appears largely unchanged (Figures 5C, 6C and 7C), while H3K9 acetylation tracks gene activation for *TOX* and *RASGRP1* but not for *NOTCH2* (Figures 5E, 6E and 7E). This pattern of repressed genes being marked with both H3K27 and H3K4 trimethylation, which we also observed on the *p16^INK4A^* promoter [11], is termed a ‘bivalent’ or ‘poised’ chromatin domain and has been identified in association with developmentally regulated genes in embryonic stem cells [60]. It will be intriguing to establish the extent to which EBNA3-regulated genes are epigenetically modified, whether the poised epigenetic state is typical, and whether genes that require the EBNA3s for their activation by EBV share this modification pattern or exhibit a distinct epigenetic mark.

Since we are examining the steady-state expression level of EBV genes, it is difficult to establish whether the differentially regulated genes are direct EBNA3 targets or are altered by indirect effects. Based on our examinations so far, we can hypothesize that genes repressed by the EBNA3s are likely characterised by H3K27 trimethylation, at least where combinations involving EBNA3C are involved. However, EBV activated genes and those apparently requiring single EBNA3s for their regulation remain untested. Global exploration of the epigenome of these cells should help clarify the regulatory mechanisms of the EBNA3s.

While EBV-infected BL31 and LCL cells both resemble activated blasts, they have approached this phenotype from opposite ends of the germinal centre reaction. This reprogramming of differentiation state is evident in BL31 both in the alteration of many transcription factors (Figure 4; Table S3) and the enrichment for germinal centre transit genes (Table S4). For instance, EBNA3B and EBNA3C appear to be essential for the repression of the key germinal centre transcription factor *BCL6*, of its co-repressor and interaction partner *BACH2* [61], and of *NFATC2* in EBV-infected BL31 cells, with mutants showing over 10-fold increase in expression. *BACH2* is repressed upon infection of naïve B cells [62], and is derepressed in 3AmutB (but not 3BKO) LCLs. Notably, genes whose expression change in different directions in 3BKO-BL31 and -LCLs also differ in expression between naïve B-cells and centroblasts. This hints that EBNA3B may be manipulating some aspects of the germinal centre transition, which is central to the *in vivo* latency program of EBV [4], [6].

The most striking functional correlation from the array data was the subtle but widespread and highly significant repressive effect of EBNA3C on genes associated with mitosis (Figure 3, Table S2). EBNA3C over-expression is known to be able to deregulate the cell cycle, overcoming checkpoints, resulting in aberrant mitosis and cytokinesis [28], [30], [32], [63]. EBV itself has been shown to protect infected cells from drugs that induce cell cycle arrest in mitotic metaphase [42]. Perhaps this alteration in the balance of mitotic proteins is a subtle manifestation of these effects.

Given the unimportance of EBNA3B in the immortalisation process, coupled to the large number of genes whose expression it appears to alter, it is likely that it has a crucial function *in vivo*, such as mobilizing the infected cell to the germinal centre or once there for the process of germinal centre transition, and perhaps the associated switch from the latency III to latency II gene expression program of EBV. Since the germinal centre process is mediated by cell-cell contact and communication, it is notable that a large number of chemokine (CCL20, CCL25, CXCL10, IL10, IL19), chemokine receptor (*CXCR7*, *CCR4*, *CCR*, *IL6R* and previously identified *CXCL4* [13]) and integrin (*ITGB1*, *ITGB3, ITGBL1* and *ITGAL*) genes significantly altered in 3BKO LCLs.

Aside from the 3Amut LCLs that we have included in our analysis, only two previous microarrays studying EBNA3 gene regulation have been published. Our analysis in IMR-90 fibroblasts (which convinced us of the need to study EBNA3s in the context of virus infection) identified a selection of chaperones and their regulator *BAG3* as EBNA3A targets [41]. Refreshingly, *BAG3* expression is also altered in 3BKO and 3AmutB LCLs . The more relevant study used an insertional mutant of EBNA3B in the EBV-BAC to generate an EBNA3B-knockout LCL [13] that also exhibited compromised EBNA3C expression in the resulting LCLs (3B^-^/3C^low^ LCLs ). This disparity was used to attribute gene regulation to EBNA3B or EBNA3C. Our 3BKO LCLs express normal EBNA3C levels (Figure S1) but deletion of EBNA3B alters the regulation of all but one (*JAG1*) of these genes, albeit a relatively small effect for *ITGA4* and *FLNA* (summarised in Table S9). That is not to say that EBNA3C plays no role in the regulation of *TCL1A* or *NCALD* – the overriding finding of our study is that many genes require multiple EBNA3 proteins for their regulation, and Hertle *et al.* previously observed that *CXCR4*, *NCALD* and *ENTH* (*CLINT1*) are differentially regulated in both EBNA3A mutant and 3B/3C^low^ LCLs [10]. We have identified 30 additional genes that appear to require both EBNA3A and EBNA3B (Table 1), although relaxing significance thresholds reveals further potential examples that could be tested by analysis of additional cell lines. In BL31 few (if any) genes require only EBNA3A and EBNA3B for their regulation, so we would predict that EBV's regulation of most of these genes will also require EBNA3C.

The comparison between genes regulated by EBNA3B and EBNA3A in BL31 and LCLs shows a rather limited set of genes to be differentially regulated in both systems (Figures 8 & 10), with only about 25% of genes altered in LCLs changed in the corresponding BL31 cell. This may be a consequence (as detailed above) of the underlying mutations that drove the original transformation and immortalisation of the parental BL31 cell line, clonal selection pressures associated with establishing LCLs, and the alternative differentiation states of the two cell types. However, where genes are regulated in both cell types the BL31 data can predict features required for their mechanism of regulation in LCLs. For instance, 14 of the 21 genes up-regulated in both 3AmutB and 3BKO LCLs are also regulated by EBNA3s in BL31 (Table 1), and this allows us to predict that *CASP8*, *CDH1*, *FLOT1*, *SLC23A2*, and *WWC3* are likely to also require EBNA3C for their repression in LCLs, while *ADARB1*, *BAG3, CREB5*, *HHEX* and *LILRA4* will not. The BL31 data also support the opposed effect of EBNA3A and EBNA3B on expression of *ST3GAL5* and *PTAFR*, as the same direction of change is seen in both LCL and BL31, supporting the statistical confidence for expression of these genes, and further illustrating the complex nature of the interactions between the EBNA3s and the host cell transcriptome.

As well as looking at the gene expression data as a whole, combining the BL31 data with the LCL data identifies a number of genes of interest (Tables 1, S6 and S8). For instance, *TCL1A* has been suggested as a key EBNA3C-regulated transforming gene [64], but we find it dispensable in 3BKO LCLs. We have also observed the expression level to increase in both wtBAC and 3BKO LCLs as the cells age (data not shown), supporting other evidence for its role in culture adaptation of LCLs rather than transformation [51]. In contrast, EBV suppresses *TERT* (the protein component of telomerase) in both BL31 and LCLs, with some evidence for a role for all of the EBNA3s in its repression. Up-regulation of telomerase appears important for the true immortalisation of transformed lymphocytes [65]. Given that primary B-cells normally express only low levels of *TERT* [66], we suggest that the EBNA3s are suppressing the action of a second factor that up-regulates *TERT*, such as LMP1 [67], [68] or c-Myc [69]. *NOTCH2* is central to the development of marginal zone (IgM^+^IgD^+^CD27^+^) B-cells [70], and its repression by EBNA3A and EBNA3C (Figure 6) may be the reason that EBV absent from this part of the memory B cell pool *in vivo* [71].

The co-operation between EBV genes extends beyond the EBNA3s. Interactome studies suggest that interaction between EBV proteins (and those of other herpesviruses) is the norm [58], [72]. But despite knowing that EBNA-LP and EBNA2 co-operate in transcriptional regulation [73], [74], [75] (and EBNA3A can interact with both [18], [58]), even the EBNA2 transactivation studies in an EBV-positive background lack a wild-type EBNA-LP [76], which seems an unfortunate oversight. Both EBNA2 and the EBNA3s can interact with DNA through their common binding partner CBF1 (RBPJκ), with the over-expression of the EBNA3 proteins able to repress EBNA2-driven activation [16], [17], [18], [77], [78]. Whether this phenomenon also occurs in EBV-infected cells is not known. Some of the genes that are derepressed in 3BKO LCLs (*CXCR7*, *TESC*, *CCR7*, *CDH1* and *ASCL1*) are induced by EBNA2-ER reactivation in EREB cells [52], [53]. In contrast, *RAPGEF*2 which is up-regulated by EBNA2-ER in the absence of CBF1 [54] also requires EBNA3s for its up-regulation by EBV in BL31 cells (and is not increased in 3BKO LCLs (Tables S1 and S5). This implies that EBNA2 and the EBNA3s may co-operate in *RAPGEF2* up-regulation, suggesting a model whereby EBNA2 target genes not requiring CBF1 for their modulation might exhibit co-operation rather than competition with EBNA3s.

There are undoubtedly additional observations that could be extracted from further study of these microarray data. Above all, we suggest that the interconnected nature of virus gene function makes it imperative that, where possible, gene expression studies should take place in the context of the whole viral genome. The EBNA3s appear to be central to the regulation of host gene expression by EBV, being involved in altering at least half of the genes regulated by EBV in BL31 cells, and that H3K27 trimethylation by the polycomb complex is central to the repression of genes by the EBNA3s. Our global approach supports a role for the EBNA3s in altering apoptotic potential and cell differentiation state, and suggests that EBNA3C subtly changes the balance of mitotic gene expression. EBNA3B is shown to be a surprisingly important contributor to the modulation of host genes. The microarray data suggest a number of high quality candidates for the mediation of EBV's effects, exploration of which should lead to the further elucidation of EBNA3 function, while further global studies are required to untangle the way in which complex interactions between the EBV gene products manipulate the host cell.

## Materials and Methods

### Generation of recombinant EBV, EBV-positive cell lines and cell culture conditions

The EBNA3A, EBNA3B and EBNA3C-exon2 deleted viruses (herein named respectively 3AKO, 3BKO and 3CKO), and their revertants (respectively 3Arev, 3Brev and 3Crev), have been described previously [42]. We have additionally generated a mutant EBV lacking all three EBNA3 genes (E3KO) and its revertant (E3rev) using previously described methods [79]. This corresponds to a deletion starting at the same point as in 3AKO and ending at the end of the 3CKO deletion - positions 92310–100998 of the B95-8 strain of EBV (Accession No V01555). These EBV-BACs were transfected into HEK-293 cells (ATCC, CRL-1573), grown in RPMI supplemented with 10% FCS, and selected in 150 µg/ml Hygromycin B. All BAC constructs have been validated by pulsed field gel electrophoresis: *Eco*RI and *No*tI digests showed the parental EBV-B95-8 BAC (wtBAC) and all the recombinants contain 6.6 copies of the BamW repeat, both in the parental BAC and in episomes rescued from the producer cell line ([42] and data not shown).

Transfection of 293 producer clones with BALF4 and BZLF1 expression plasmids produced infectious recombinant viruses as described previously [80]. Virus from independently generated 293 producer clones was used to infect the EBV-negative Burkitt's lymphoma cell line BL31 [81]. Newly infected BL31 cell lines were grown out and propagated in RPMI supplemented with 1mM sodium pyruvate, 50 µM thioglycerol, 10% FCS, penicillin and streptomycin and selected with 200 µg/ml Hygromycin B. Once established, Hygromycin B concentration was reduced to 125 µg/ml. Previously described BL31 cells were grown in RPMI supplemented with 10% FCS, penicillin and streptomycin. BL31 cell lines were split 1∶3 twice a week to maintain growth in culture.

To generate LCLs using B95-8-BAC (wtBAC) and EBNA3B knockout (3BKO) viruses, we infected 10^6^ peripheral blood leukocytes (PBLs) with 100 µl of virus preparation (at between 50 and 300 Raji-green-units per microlitre). The PBLs for generating the LCLs came from three EBV-negative blood donors (kind gift from Dr Ingo Johannessen, University of Edinburgh) designated D1, D2 and D3. For each donor, PBLs were infected with virus from two independent HEK-293 cell lines carrying the parental B95-8 BAC and from two 3BKO producer lines carrying the EBNA3B knockout BAC (3BKO). LCLs were grown out from these infections in RPMI supplemented with 15% FCS, and once sufficient cells had been established (typically 3–6 weeks post infection), they were seeded at 5×10^5^ cells per ml. After 24 hours, 15 ml of cells were harvested for RNA isolation.

### Microarray analysis

BL31 cell lines were grown for 48 hours after splitting 1∶3 and then RNA was extracted from 30 ml of cell culture using a Qiagen RNeasy Midi kit according to manufacturer's protocol. Where RNA was less than 500 ng/µl it was concentrated by precipitation with sodium acetate and ethanol. LCLs were seeded at 5×10^5^ cells per ml,15 ml harvested 24 hours later and RNA isolated using Qiagen RNeasy mini kit as instructed. RNA was quantified by NanoDrop spectrophotometer (Thermo Scientific) and checked for integrity on a Bioanalyzer (Agilent). RNA was labelled using the GeneChip Affymetrix whole transcript sense target labelling assay. Essentially this comprises a ribosomal RNA reduction step using the Ribominus human transcriptome isolation kit (Invitrogen), and generation of labelled sense-strand cDNA. This was then hybridised to an Affymetrix Human Exon 1.0ST array and scanned.

Array data was extracted using Expression Console software to generate CEL files. CEL files were summarised with MMBGX (Multi-Mapping Bayesian Gene eXpression) v0.99.5 using the X:Map/Ensembl database for Homo Sapiens (GRCh37) v56 (xmap_homo_sapiens_core_56_37a.tgz). MMBGX uses X:Map mappings between probes and transcripts encoded in Ensembl cDNA sequences to generate Exon array probesets [46]. MicroRNA sequences are not included in the cDNA tables, but are mapped to the genome by X:Map. Therefore we created additional probesets targeting microRNAs and included these in our analysis. Mean posterior expression estimates for each gene were log2 transformed. The CEL files and gene level MMBGX estimates are deposited in the EBI ArrayExpress database (http://www.ebi.ac.uk/microarray-as/ae/) with accession ID E-MEXP-2767 for the BL31 data and E-MEXP-2768 for 3BKO and wtBAC LCLs. Processed microarray data from the EBNA3A LCLs (deposited at EBI Array Express with accession ID E-GEOD-17908)[10]) was log2 transformed before further analysis and annotated according to the Ensembl v56 annotation of the probesets obtained using Biomart, to facilitate comparison with the exon array data.

Subsequent analyses (principle component analysis, ANOVA, false discovery rate calculation, clustering etc) were performed using Partek Genomic Suite version 6.4 or 6.5 except where stated otherwise. Generally, statistical analyses were performed using the default settings. Hierarchical clustering was performed using average linkage and Euclidian dissimilarity in a 2-pass method.

Differentially regulated genes were defined by ANOVA. In order to establish the best model for experimental and batch effects, a series of ANOVAs were used to assess the contribution of different sources of error (potential causes of batch effects are described in the array annotation deposited with the raw microarray data). Essentially, the model that best fit the data (i.e. the error was the smallest contributor to the source of variation in the system) was a 3-way ANOVA incorporating the media type in which the cells were grown and the microarray lot number used for the sample. False discovery rate analyses on the p-values of factors in the various ANOVAs confirmed that other factors caused no more variation than chance.

Differentially regulated genes in 3Amut LCLs and 3BKO LCLs were identified using an ANOVA model including the donor ID (for genetic background of the cells). For comparison of 3BKO and 3Amut LCLs, the Exon array gene list was reduced to include only genes represented on the U133 array (see Supporting Information File S1 for details). For comparison, the genes differentially regulated between 3BmutB and wtBAC were used, as 3AmutB is the most similar to our 3AKO virus.

### Gene Ontology

Grouping for ontologically similar genes was assessed using the Database for Annotation, Visualization and Integrated Discovery (DAVID) version 6.7. Specifically, for each contrast between an EBNA3 knockout and the wild-type-infected cells, lists of genes were generated whose members passed one of the following threshold combinations: p<0.0001 and fold change >1.3 or p<0.001 and fold change >2 or p<0.01 fold change >3. These were analysed for up and down-regulated genes both separately and together, and functional annotation groups were identified both with and without clustering (at medium stringency). The significance of any ontological label is presented either as false discovery rate q-value according to Benjamini (from the DAVID functional annotation chart) or as a group enrichment score (the negative log of the geometric mean of the group's members' EASE p-values - tables S2 and S7) from the functional annotation clustering.

### Western Blot analysis and generation of anti-EBNA3B antibody 6C9

Western blots were performed using minigels (Bio-Rad) as described previously [42]. Primary antibodies used were as follows: mouse monoclonal antibodies against EBNA2 (clone PE2; DAKO), LMP1 (CS1-4; DAKO), EBNA3C (A-10: hybridoma a gift of Martin Rowe), EBNA-LP (JF186 [82]), gamma-tubulin (GTU-88, Sigma). Sheep polyclonal antibody against EBNA3A (ExAlpha, Maynard MA USA).

For detection of EBNA3B, we have raised a rat monoclonal antibody (clone 6C9) against amino-acids 328–624 of EBNA3B fused to GST. A HincII/MseI fragment of of the EBNA3B cDNA was ligated in-frame into the pGEX-3X GST-fusion vector. The resulting fusion protein was purified from lysates of *E. Coli* on glutathione-sepharose beads and used for immunisation of rats as described previously [83].

### Real-time quantitative PCR (qPCR)

Validation of the microarray data was achieved using a custom Taqman Low Density Array card (Applied Biosystems). The assays used are summarised in Table S10. Each 48 assay channel was loaded with 1 µl of cDNA (generated from 750 µg of total RNA using the SuperScript® III First-Strand Synthesis Supermix, Invitrogen) in TaqMan® Gene Expression Master Mix (Applied Biosystems). qRT-PCR was performed on a 7900HT fast real-time system (Applied Biosystems) using recommended conditions, and Ct values extracted using SDS2.3. Five reference genes were included on the array card (*ACTB*, *GAPDH*, *GNB2L1, GUSB*, *HMBS*) and were used to generate normalisation factors for each sample using qBase Plus v1.4 (Biogazelle, Ghent, Netherlands) which were used to normalise the Ct values. Relative quantities were generated assuming an assay efficiency of 100% and assigning a value of 1 to a normalised Ct of 40 (ie no qPCR amplification). These quantities were also analysed by ANOVA, applying the same model as used for the microarray.

### Chromatin immunoprecipitation (ChIP) assay

ChIP assays were carried out as described previously [26] using a ChIP Assay Kit (17–295; Millipore) according to the manufacturer's instructions. Chromatin was sheared to a range of 200bp – 800bp in length from 1×10^6^ cells per ChIP in 200 µl of lysis buffer using a Bioruptor sonicator (UCD-200; Diagenode) on a high setting for a total of 12 min (30 sec ‘on’/30 sec ‘off’ intermittent sonication). Chromatin was precipitated using anti-H3K27me3 antibody (ChIPAb+ 17-622; Millipore) and, as a negative control, rabbit IgG serum (PP64B; Millipore) was used. Isolated DNA was assayed by quantitative PCR (qPCR) using the Platinum SYBR Green qPCR SuperMix (11733; Invitrogen) and an ABI 7900 384-well Real-Time PCR machine. The sequence of primers used for each promoter specific PCR are attached in Table S11. Cycling conditions used were: 2 min at 50°C, 10 min at 95°C and then 15 sec at 95°C, 1 min at 60°C for 40 cycles. Using standard curves, 1% of input was compared to the IP sample and the values from the IgG negative control were subtracted as background. The data are representative of two independent experiments and are given as means ± 1SD for triplicate PCRs.

## Supporting Information

File S1 Gene set enrichment analysis shows EBNA3s alter genes involved in the germinal centre transition. Analysis of EBNA3A-mutant LCL U133 microarray data. Transcript-level analysis of the microarray is only indicative of differential transcript use.
(0.06 MB DOC)

Figure S1 EBV latent gene expression in BL31 cell lines is not altered by EBNA3 deletion. Western blots for EBV latent proteins expressed in the newly described cell lines are shown here. As shown previously EBNA3 deletion does not consistently alter latent gene expression [48].
(1.15 MB PDF)

Figure S2 Gene expression data clusters cell lines according to the EBV mutant (A) 3-dimensional principle component analysis of raw gene level expression values. Colour indicates the virus type used and the shape indicates the growth media used. (B) Dendrogram generated by unsupervised clustering of cell lines based on the expression values of the subset identified as differentially regulated (p<0.005; Fold change >2) between EBV-negative BL31 and wild-type-infected BL31. Dendrogram line colours match virus type in the PCA. Block colours further distinguish the virus used (i.e. separate the different revertants within the wtBAC group).
(0.53 MB PDF)

Figure S3 Protein levels of EBV latent genes are unaffected by absence of EBN3B in LCLs. Western blots of the major EBV latent proteins were performed on protein samples taken at the same time as the RNA for the microarray. EBNA3A and LMP2A westerns are on total protein extracts (cells lysed in SDS sample buffer) while the others are from RIPA protein extracts.
(0.56 MB PDF)

Figure S4 Validation of 3BKO LCL microarray expression level data by qPCR. Log2 transformed gene expression values for 42 genes for the six 3BKO LCLs and six wtBAC LCLs were established by qPCR using the same RNA samples that were used for the microarrays. These are plotted against the corresponding gene level expression value from the MMBGX microarray analysis. Correlation coefficient and best fit line are indicated. Assays deviating from the trendline (GAPDH, ACTB, SOX4 and TERT) are coloured as indicated.
(0.07 MB PDF)

Figure S5 qPCR assessment of transcript-level microarray analysis. As candidates for assessing the transcript level analysis of gene expression, we have looked at TERT (A-D), TSC22D1 (E) and TNFSF10 (F) expression in 3BKO and wtBAC LCLs. In each case the same RNA samples were used for the qPCR as for the microarray. (A) A graphical representation (from xmap.picr.man.ac.uk) of the TERT transcript variants in ENSEMBL release 56. Locations of probes from the Exon microarray are indicated by vertical green lines (where they map uniquely to the genome) and grey lines (mapping to multiple genome locations). Exons are indicated by red boxes, and the exon junctions studied are shown. (B-F) Box and whiskers plots, indicating median, and 25th and 75th percentile values (box) with whiskers indicating minimum and maximum values). Y-axis scale is a log 2 gene expression level scale, taken from the microarray or from a modified 2-delCt calculation (see methods). For TERT, (B) shows transcript-level output of the MMBGX algorithm, (C) sums the appropriate transcripts to estimate exon-exon junction levels and (D) is quantitation of these exon-exon junctions by qPCR. Similarly, (E) and (F) show the estimated exon-exon junction quantities from transcript level analysis (left - marked ‘Array’) and qPCR analysis of these junctions (right) for TSC22D1 and TNFSF10 respectively.
(0.71 MB PDF)

Table S1 BL31 gene level ANOVA statistics. Complete list of all genes with contrast statistics showing p-value and fold change of mutant EBV-infected cell lines as compared to wtBAC-infected. Also includes uninfected BL31 compared to wtBAC. Negative fold change indicates lower expression level in mutant compared to wtBAC cells.
(9.43 MB XLS)

Table S2 Functional groups altered by the deletion of EBNA3s in BL31. Cluster enrichment scores (from DAVID) for lists of genes regulated by each single EBNA3 knockout. For each EBNA3 knockout, statistics are shown for up-regulated and down regulated genes independently, and for these two lists combined. The value is a negative log10 of the enrichment p-value for the combined set of ontology groups indicated (ie high values are more significant).
(0.04 MB XLSX)

Table S3 ANOVA statistics for major players in B-cell differentiation. Contrast statistics showing statistical significance and fold change for genes shown in Figure 4. Emphasis (bold and/or colour) is placed where statistics pass certain thresholds as indicated in the table.
(0.06 MB XLSX)

Table S4 Gene set enrichment analysis of EBV and EBNA3-regulated genes for genes involved with the germinal centre transition. Statistics assessing enrichment (skewing) of total exon array data for genes differentially expressed between germinal centre and naive and memory B cells, according to Klein et al 2003 [58]. Table shows GSEA enrichment score (NES) and q-value (False discovery rate for chance enrichment of the gene list). Blue figures indicates a positive correlation (more than a random number of these these genes are expressed at a higher level in wtBAC than the mutant/uninfected BL31 cells), red figures a negative correlation (enrichment for genes expressed higher in the mutant). The transition analysed is indicated at the top of the table, and up- and down-regulated genes are treated separately. Emphasis (colour; bold) thresholds are indicated below the table.
(0.07 MB XLSX)

Table S5 ANOVA statistics indicating differential gene expression between wtBAC and 3BKO LCLs. Whole genome gene-level analysis fold change and significance for each gene. Positive value indicates higher expression in 3BKO LCLs. The Donor effect p-value indicates whether the expression level is significantly affected by the different genetic background giving rise to the LCLs. Gene list is sorted from most significantly different gene.
(2.36 MB XLSX)

Table S6 ANOVA statistics for genes differentially regulated by EBNA3B in both BL31 and LCLs. List of genes significantly altered by EBNA3B deletion (p<0.001 and fold change >2) in both BL31 cells and LCLs. Table is separated into three sections (up-regulated in 3BKO cells, down-regulated, and up in 3BKO BL31 but down in 3BKO LCLs (as per the numbers indicated in the venn diagram in Figure 8). Genes are listed alphabetically by name in each section. ANOVA statistics showing the effects of other EBNA3 mutants in BL31 are included for interest.
(0.06 MB XLSX)

Table S7 Gene ontology cluster enrichment for 3BKO LCLs. Cluster enrichment scores for the 3BKO-LCL differentially expressed genes are indicated (see Table S2 for more details).
(0.04 MB XLSX)

Table S8 ANOVA statistics for genes differentially regulated by EBNA3A in both BL31 and LCLs. List of genes significantly altered by EBNA3A deletion (p<0.001 and fold change >2) in both 3AKO-BL31 cells and 3AmutB-LCLs (according to Hertle et al 2009). Table is separated into three sections (down-regulated in 3AKO cells, up-regulated, and up in 3AKO BL31 but down in 3AmutB LCLs (as per the numbers indicated in the venn diagram in Figure 10A). Three additional genes are included where 3AmutB statistics marginally failed to pass the significance or fold-change threshold, but when all 3Amut LCLs were included, they did pass these thresholds. Statistics for all probesets in the U133 microarray mapping to the gene (according to Ensembl genome annotation v56) are included. Those passing significance are indicated in bold. Green indicates up-regulated in the mutant, red indicates expressed at lower level. Genes are listed alphabetically by name in each section.
(0.06 MB XLSX)

Table S9 Genes previously characterised in EBNA3B-knockout LCLs. Fold changes previously described by Chen et al (2006) for 3B-3Clow LCLs by qPCR and U133 microarray are compared to gene level changes from our 3BKO LCLs vs wtBAC-LCLs in our Exon microarray. The highly significantly regulated genes (p<0.001) by microarray are also tested by qPCR, both on the same RNA and on an independent RNA sample. qPCR fold changes assume assays are 100% efficient. Genes whose names have changed since Chen et al (2006) are CLINT1 (formerly ENTH), PIK3R5 (formerly P101-PI3K) ARL6IP5 (formerly JWA) and TIAM1 (formerly U90902.1).
(0.05 MB XLSX)

Table S10 Identities of qPCR assays used to test gene expression. All assays were used in Taqman low density array cards (Applied Biosystems). Top array set was used for assessing reliability of the microarrays (Figure 2 and S4). Lower list are additional assays used to assess reliability of transctipt-level microarray analysis (supporting information and Figure S5) Assay details (including map of where in the gene they map are available from the applied biosystems website.
(0.05 MB XLSX)

Table S11 Primers used for qPCR analysis of ChIP samples to determine histone modification association at cellular promoters. Position of the assay relative to transcription start site (-, upstream; + downstream) is indicated. Assay identifier (number -3 to +2) is as used in Figures 5-8. ‘p’, promoter.
(0.05 MB XLSX)
