# Supplementary material for: Extensive Co-Operation between the Epstein-Barr Virus EBNA3 Proteins in the Manipulation of Host Gene Expression and Epigenetic Chromatin Modification
Source: PLoS One. 2010 Nov 15;5(11):e13979. doi: 10.1371/journal.pone.0013979 (PMC2981562; doi:10.1371/journal.pone.0013979)
Supplement: File S1 — Gene set enrichment analysis shows EBNA3s alter genes involved in the germinal centre transition. Analysis of EBNA3A-mutant LCL U133 microarray data. Transcript-level analysis of the microarray is only indicative of differential transcript use. (0.06 MB DOC) [file pone.0013979.s001.doc]

**Supporting Information**

**Gene set enrichment analysis shows EBNA3s alter genes involved in the germinal centre transition.**

Klein *et al* performed an extensive microarray experiment on B-cells from the different differentiation states through the germinal centre transition, namely naive B cells, centroblasts, centrocytes and memory B cells [1]. They observed that the gene expression difference between naive B cells and memory B cells (ie before and after germinal centre) was fairly modest (approx 50 genes altered), and the difference between centroblast and centrocyte (within the germinal centre) was even more slight (just 18 genes). However, a much larger number of genes differentiated the germinal centre transcriptome from the pre/post GC profile, many of which are proliferation-associated.

We split the lists of genes altered at each transition (from Klein *et al* [1] supplementary figure 6) through the germinal centre (according to whether they were up- or down-regulated at that transition) and uploaded the log2 transformed MMBGX gene level expression data to the Gene set enrichment analysis (GSEA) client. Gene lists were ranked by GSEA for each contrast, comparing the mutant-infected to wtBAC-infected cells. Positive or negative enrichment of these lists for members of the gene sets up and down regulated in the germinal centre transitions (compared to 1000 random ranked lists) and enrichment statistics (NES and false discovery rate q value) are shown in Table S4)

Where the enrichment score (NES) is positive (blue), this indicates a positive correlation between the gene lists (ie genes at the top of the ranked list - up-regulated in the mutant as compared to the wild-type - are enriched in the germinal centre list). Negative enrichment (red) indicates that the bottom of the list (down-regulated genes) is enriched for members of the germinal centre list. Thus we see that EBV negative BL31 cells express higher levels of genes up-regulated in the naive to GC transition, and lower levels of genes repressed during this transition. The inverse is true of the centrocyte to memory transition (although many of the genes in the ‘down’ list for N to CB are also in the CC to M transition) but in all suggests that the BL31 cells have some germinal centre characteristics that are being changed to a naive/memory cell characteristic upon infection by EBV and adoption of the latency III gene expression pattern. The EBNA3 gene expression is undoubtedly a major player in this change, as the altered genes in E3KO matches the BL31 pattern well. Of the single gene mutants, EBNA3C appears to be involved in altering these genes, while EBNA3B-regulated genes are enriched for genes expressed at a higher level in the germinal centre, but not for genes expressed at a lower level. The GSEA enrichment plot, and examination of the differentially regulated genes indicates that this is not so much a consequence of fewer genes being down-regulated in 3BKO-BL31s, but rather because this is offset by the up-regulation (compared to wtBAC-BL31) of additional genes.

**Analysis of EBNA3A-mutant LCL U133 microarray data.**

In order to compare the published microarray analysis of EBNA3A mutant LCLs [2] with our 3BKO LCLs, the ENSEMBL ID for each probeset ID on the Affymetrix U133v2.0 microarray was extracted from the ENSEMBL v56 genome release (Human genes GRCh37) using BIOMART (http://www.ensembl.org/biomart/martview). This was used to re-annotate the processed microarray data (EBI Array Express ID: E-GEOD-17908). Of the 22227 human probesets on the U133plus2 chip, 20,056 were represented in the ENSEMBL genome annotation, and of these 18227 had a single ENSEMBL ID, with 12056 different genes represented. The data were statistically analysed by ANOVA using the virus type (wtBAC, 3AmutA and 3AmutB) and cell donor ID as factors. We have included genetic background as, despite the lack of a balanced distribution of genetic backgrounds between the groups, visualisation of individual datasets suggests that this imbalance does not detectably distort the statistical analysis. To compare Exon and U133 arrays, the 3AKO data was used where either i) the ENSEMBL gene ID(s) matched that of the Exon array ENSEMBL IDs (18024 U133plus2 probesets) or ii) where the first gene ID of U133 probesets having multiple IDs maps to an Exon array gene (1866 additional probesets) for a total of 19908 probesets mapping to 12863 genes on the exon array. The two mutants analysed were deletions of either the whole of the EBNA3A gene (3AmutB), very like our 3AKO mutation, and a mutant retaining the first exon and intron of EBNA3A (3AmutA) that could hypothetically express a 126 amino acid N-terminal fragment of EBNA3A. Observing that the two mutants behaved differently, with a substantial subset of genes differentially regulated between 3AmutB and wtBAC-LCLs that were not changed in mutA LCLs, comparisons with our array data were performed using the statistics from the 3AmutB contrast, as this virus is the most like our 3AKO mutant.

**Transcript-level analysis of the microarray is only indicative of differential transcript use.**

One of the advantages of the Exon microarray is the ability to analyse gene expression at the transcript level. To investigate this we tested three genes to clarify which splice variants are produced.

Telomerase reverse transcriptase (TERT) is the protein component of telomerase, and is not normally expressed in LCLs, although the permanent transformation of cell lines is typically accompanied by increased telomerase actvity (reviewed in [3]). At the gene level, *TERT* expression is low in wtBAC-LCLs and increased 2.8 fold in 3BKO-LCLs. It is similarly repressed by EBV in BL31 cells, but repression is lost in 3BKO, 3CKO and E3KO BL31s. ENSEMBL records 6 alternative isoforms for *TERT* (Figure S5A) and according to the MMBGX transcript level analysis in both BL31 and 3BKO-LCLs, the main transcript upreglated in EBV-mutant cells is isoform TERT-003 (see Figure S5B), which lacks the first two exons of the *TERT* gene, as well as exons 6 and 7 which results in a frame shift and nonsense mutation [4].

In order to test whether this isoform was the predominant one, we assayed the expression of the exon junctions indicated (Figure S5A). Comparing the exon junction frequencies predicted by the microarray (Figure S5C) with the qPCR results (Figure S5D), it is clear that the difference in *TERT* expression between 3BKO-LCLs ad wtBAC-LCLs as measured by qPCR is considerably greater than that predicted by the microarray. Indeed, most of the wtBAC-LCLs had no detectable TERT transcripts where the microarray expression profile would predict some, albeit at a low level. In contrast, qPCR of *TERT* RNA from the wtBAC-BL31 cells did reflect the microarray fairly accurately (Figure 2) although the difference between wild-type and mutant is again more pronounced than predicted. Here, *TERT* repression is lost in the 3BKO, 3CKO and E3KO-BL31 lines, but not in 3AKO or EBNA2 knockout BL31s (data not shown).

At the level of splice junctions, the reduced qPCR signal from exons 2-3 and exons 6-7 compared to the other assays supports the array observation that the predominant isoform is transcribed from exon 3, and excludes exons 6 and 7 in both BL31 and LCLs. However, there is no suggestion of the exclusion of exon 11 (also seen in TERT-003). So while there is some evidence of enrichment of a TERT-003-like transcript, the difference in levels of the qPCR assays (only 1-2 units on a log2 scale) is rather dwarfed by the difference between mutant and wild-type-infected cells (3-4 units, or >10-fold difference in BL31, and considerably more in 3BKO LCLs).

*TNFSF10* encodes the protein TRAIL, also known as CD253. TRAIL is a key player in the promotion of apoptosis, being both expressed on and cleaved from the cell surface. TRAIL is able to induce apoptosis by binding to trail receptors 1 or 2 (TNFRSF10A and B) but its effects are modulated by binding to TRAIL receptor 3 or 4 (TNFRSF10C or D). At the gene level it is expressed at lower levels in 3BKO LCLs, as well as in EBNA3A mutant LCLs as compared to wtBAC-LCLs ([2] and Table 1). ENSEMBL lists a number of transcripts (not shown) which are either short transcripts with alternative second or third exons, or full length transcripts missing exon 3. The transcript level analysis indicated a higher expression for exon 1-2 than 3-4, with higher expression of both in the wtBAC LCLs (Figure S5E). qPCR analysis of these exons confirmed the differential regulation between wtBAC and 3BKO-LCLs, but quantity of the exon1-2 and 3-4 splice junctions were indistinguishable, suggesting that the full length transcript containing all exons is the only TNFSF10 mRNA in these cells (Figure S5E).

TSC22D1 is a transcription factor induced by TGFβ signalling, and has been previously reported to be repressed during the activation of naive B cells [5]. Microarray analysis suggests a fairly low level expression of the gene in wtBAC LCLs that is reduced in 3BKO LCLs. The major transcripts of this 3 exon gene initiate either at a distal exon 1, almost 140 kb upstream of exon 2, or at a proximal exon 1b, just 5 kb away. The microarray suggests that the longer exon 1-2 transcript(s) are expressed at a higher level, while the minority exon 1b-2 transcript is more reduced in the 3BKO LCL (Figure S5F). The qPCR data supports this analysis entirely, with the minority isoform with the proxomal first exon (1b-2) being substantially reduced in 3BKO LCLs while the longer transcriptional unit is not significantly different between the two LCL types.

In summary, the data from the MMBGX transcript level analysis is supported for *TSC22D1*, partially supported for *TERT*, and not supported at all for *TNFSF10*, suggesting that transcript-level MMBGX data can offer an indication of which transcripts are being used, but is not always an accurate predictor of expression magnitude.

**Supporting References**

1. Klein U, Tu Y, Stolovitzky GA, Keller JL, Haddad J, et al. (2003) Transcriptional analysis of the B cell germinal center reaction. Proc Natl Acad Sci USA 100: 2639-2644.

2. Hertle ML, Popp C, Petermann S, Maier S, Kremmer E, et al. (2009) Differential gene expression patterns of EBV infected EBNA-3A positive and negative human B lymphocytes. PLoS Pathog 5: e1000506.

3. Sugimoto M, Tahara H, Ide T, Furuichi Y (2004) Steps involved in immortalization and tumorigenesis in human B-lymphoblastoid cell lines transformed by Epstein-Barr virus. Cancer Res 64: 3361-3364.

4. Kilian A, Bowtell DD, Abud HE, Hime GR, Venter DJ, et al. (1997) Isolation of a candidate human telomerase catalytic subunit gene, which reveals complex splicing patterns in different cell types. Human Molecular Genetics 6: 2011-2019.

5. Glynne R, Ghandour G, Rayner J, Mack DH, Goodnow CC (2000) B-lymphocyte quiescence, tolerance and activation as viewed by global gene expression profiling on microarrays. Immunol Rev 176: 216-246.
