# Supplementary figures and images for: Extensive Co-Operation between the Epstein-Barr Virus EBNA3 Proteins in the Manipulation of Host Gene Expression and Epigenetic Chromatin Modification
Source: PLoS One. 2010 Nov 15;5(11):e13979. doi: 10.1371/journal.pone.0013979 (PMC2981562; doi:10.1371/journal.pone.0013979)

Figure S1

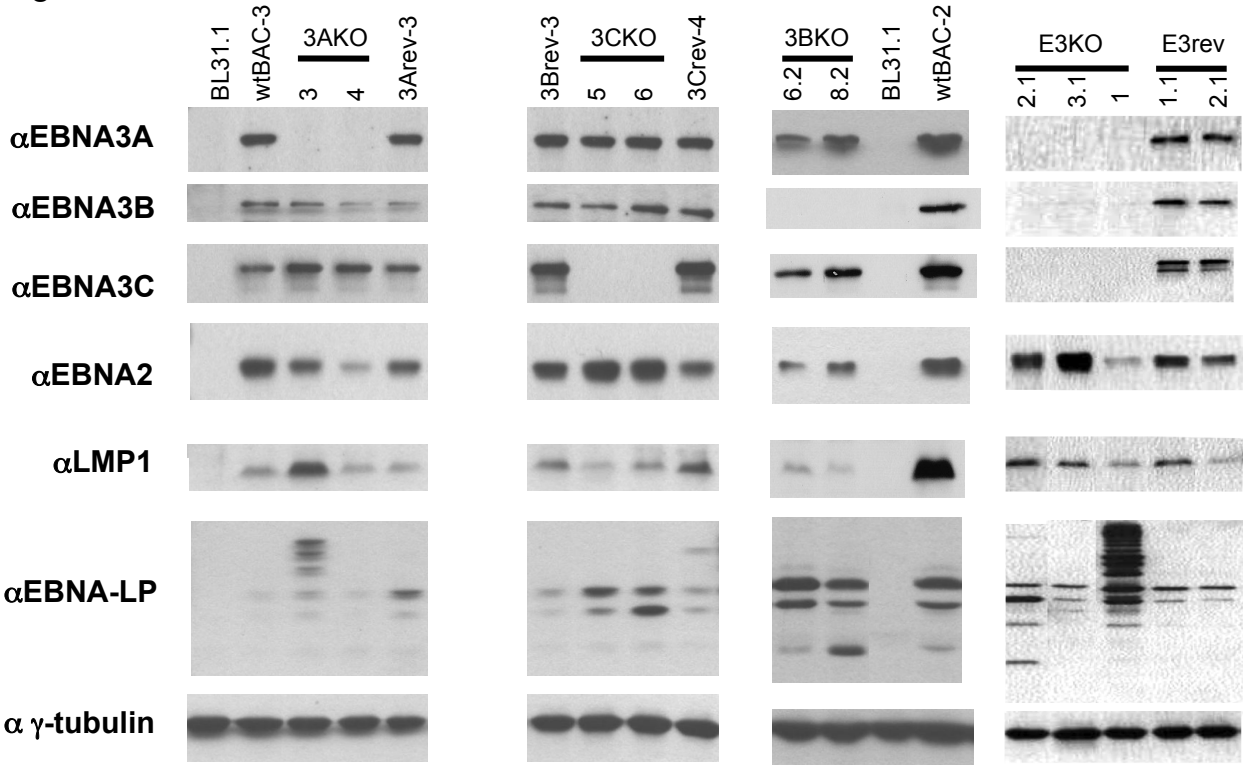

Supplement: Figure S1 — EBV latent gene expression in BL31 cell lines is not altered by EBNA3 deletion. Western blots for EBV latent proteins expressed in the newly described cell lines are shown here. As shown previously EBNA3 deletion does not consistently alter latent gene expression [48]. (1.15 MB PDF) [file pone.0013979.s002.pdf]

PCA Mapping (34.8%)

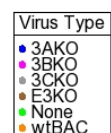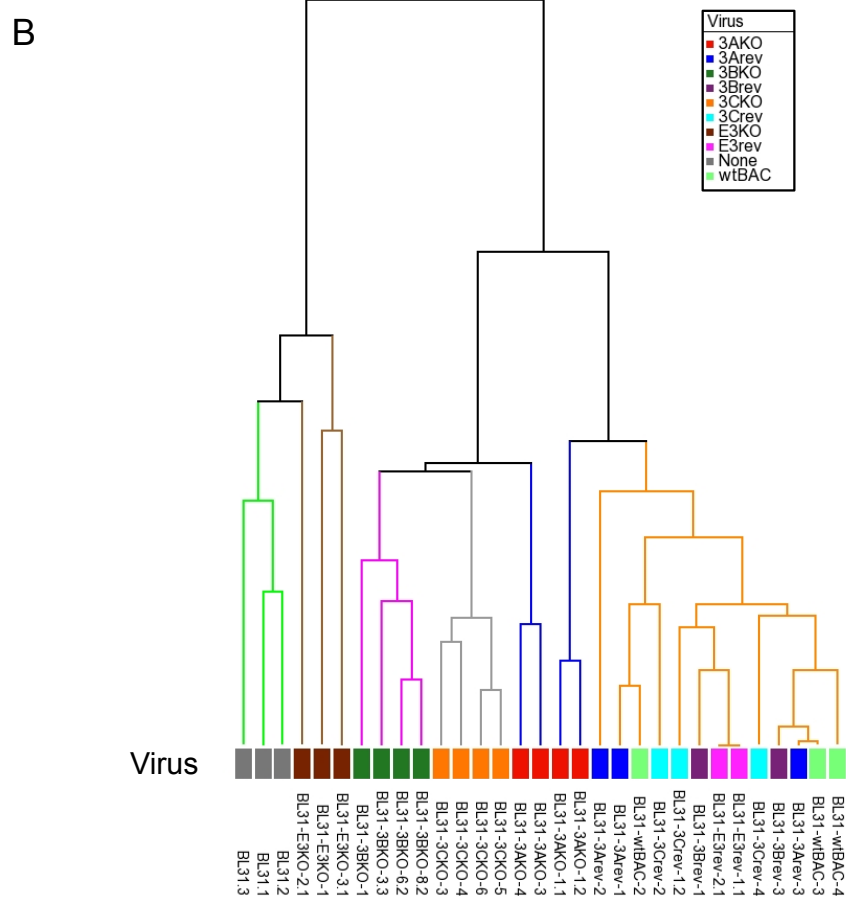

Supplement: Figure S2 — Gene expression data clusters cell lines according to the EBV mutant (A) 3-dimensional principle component analysis of raw gene level expression values. Colour indicates the virus type used and the shape indicates the growth media used. (B) Dendrogram generated by unsupervised clustering of cell lines based on the expression values of the subset identified as differentially regulated (p<0.005; Fold change >2) between EBV-negative BL31 and wild-type-infected BL31. Dendrogram line colours match virus type in the PCA. Block colours further distinguish the virus used (i.e. separate the different revertants within the wtBAC group). (0.53 MB PDF) [file pone.0013979.s003.pdf]

Figure S3

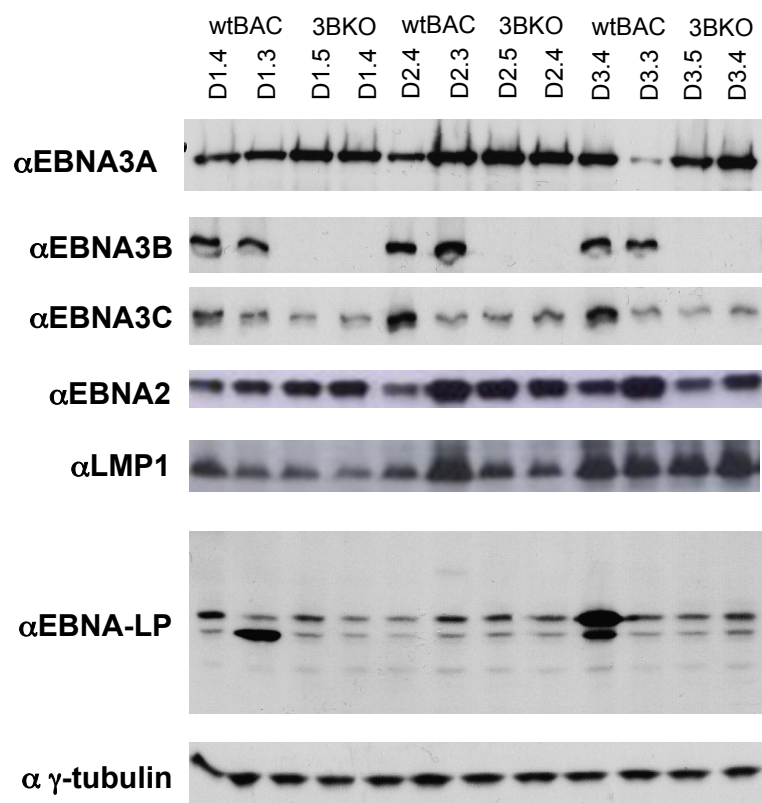

Supplement: Figure S3 — Protein levels of EBV latent genes are unaffected by absence of EBN3B in LCLs. Western blots of the major EBV latent proteins were performed on protein samples taken at the same time as the RNA for the microarray. EBNA3A and LMP2A westerns are on total protein extracts (cells lysed in SDS sample buffer) while the others are from RIPA protein extracts. (0.56 MB PDF) [file pone.0013979.s004.pdf]

Figure S4

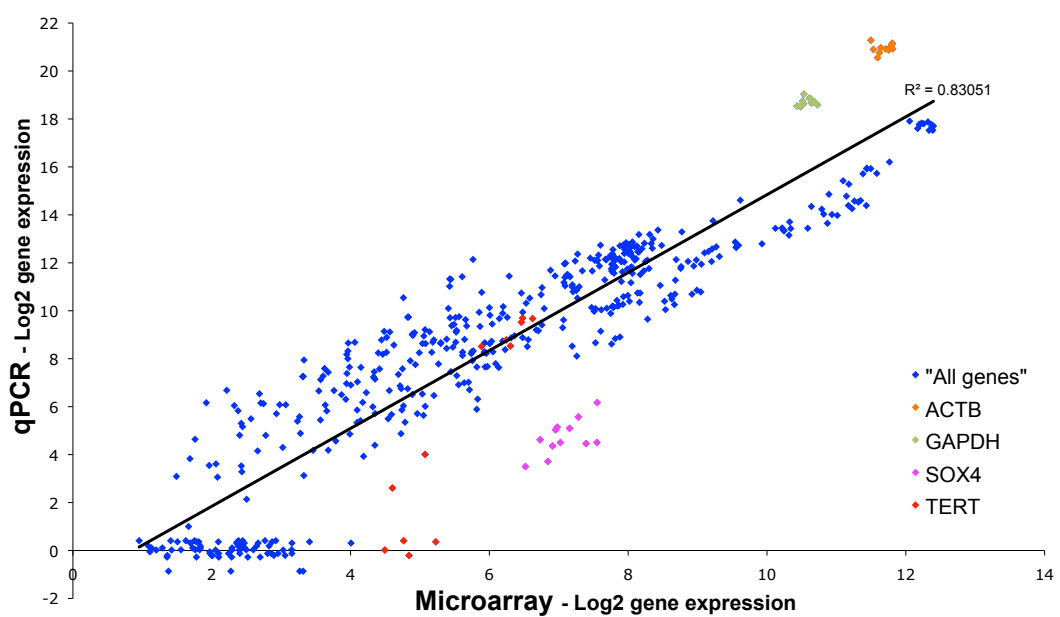

Supplement: Figure S4 — Validation of 3BKO LCL microarray expression level data by qPCR. Log2 transformed gene expression values for 42 genes for the six 3BKO LCLs and six wtBAC LCLs were established by qPCR using the same RNA samples that were used for the microarrays. These are plotted against the corresponding gene level expression value from the MMBGX microarray analysis. Correlation coefficient and best fit line are indicated. Assays deviating from the trendline (GAPDH, ACTB, SOX4 and TERT) are coloured as indicated. (0.07 MB PDF) [file pone.0013979.s005.pdf]

Figure S5

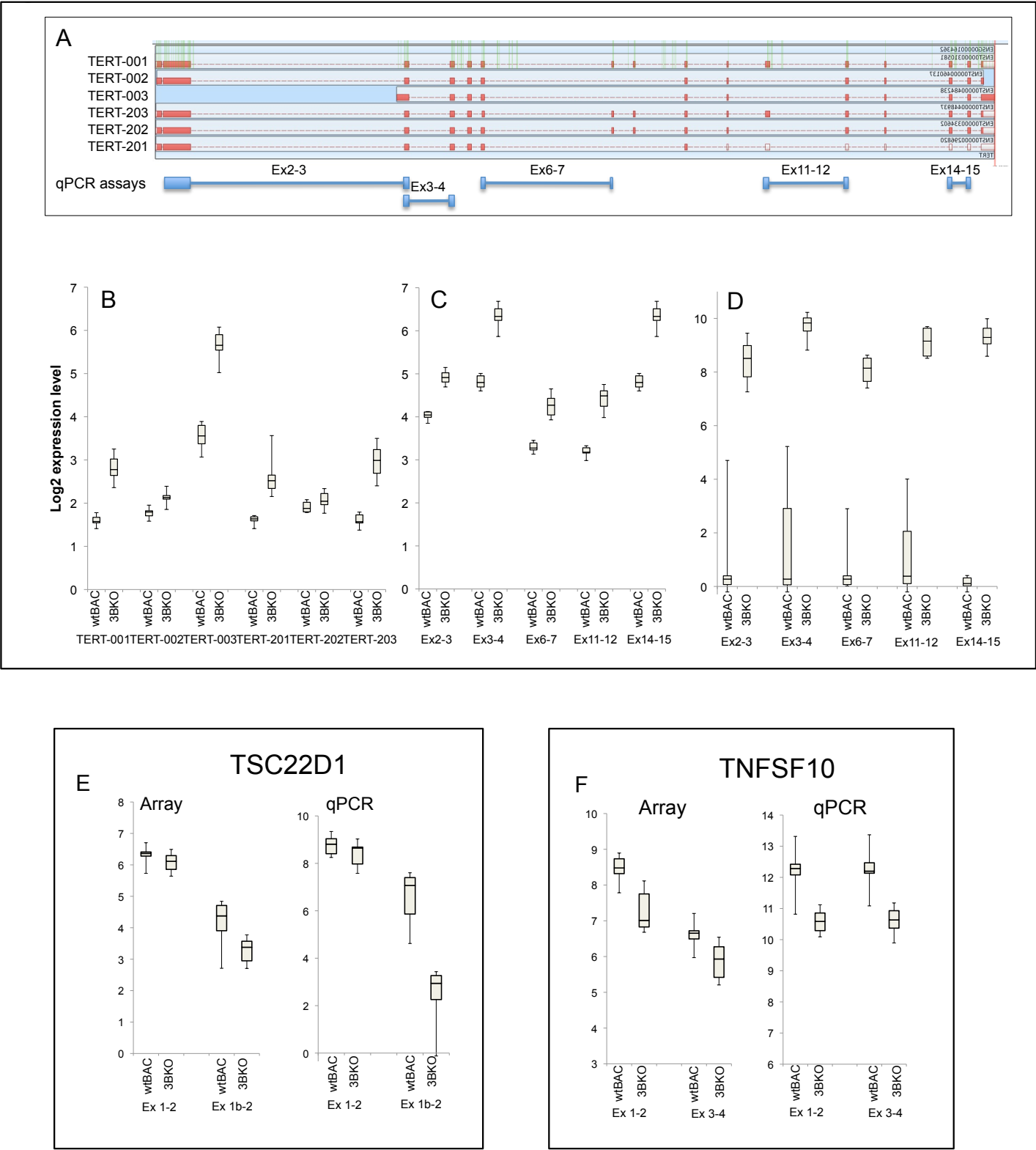

Supplement: Figure S5 — qPCR assessment of transcript-level microarray analysis. As candidates for assessing the transcript level analysis of gene expression, we have looked at TERT (A-D), TSC22D1 (E) and TNFSF10 (F) expression in 3BKO and wtBAC LCLs. In each case the same RNA samples were used for the qPCR as for the microarray. (A) A graphical representation (from xmap.picr.man.ac.uk) of the TERT transcript variants in ENSEMBL release 56. Locations of probes from the Exon microarray are indicated by vertical green lines (where they map uniquely to the genome) and grey lines (mapping to multiple genome locations). Exons are indicated by red boxes, and the exon junctions studied are shown. (B-F) Box and whiskers plots, indicating median, and 25th and 75th percentile values (box) with whiskers indicating minimum and maximum values). Y-axis scale is a log 2 gene expression level scale, taken from the microarray or from a modified 2-delCt calculation (see methods). For TERT, (B) shows transcript-level output of the MMBGX algorithm, (C) sums the appropriate transcripts to estimate exon-exon junction levels and (D) is quantitation of these exon-exon junctions by qPCR. Similarly, (E) and (F) show the estimated exon-exon junction quantities from transcript level analysis (left - marked ‘Array’) and qPCR analysis of these junctions (right) for TSC22D1 and TNFSF10 respectively. (0.71 MB PDF) [file pone.0013979.s006.pdf]
